# The PlcR Virulence Regulon of *Bacillus cereus*

**DOI:** 10.1371/journal.pone.0002793

**Published:** 2008-07-30

**Authors:** Michel Gohar, Karoline Faegri, Stéphane Perchat, Solveig Ravnum, Ole Andreas Økstad, Myriam Gominet, Anne-Brit Kolstø, Didier Lereclus

**Affiliations:** 1 INRA, Génétique microbienne et Environnement, Guyancourt, France; 2 INRA, Microbiologie et Génétique Moléculaire, Thiverval-Grignon, France; 3 Department of Pharmaceutical Biosciences, University of Oslo, Oslo, Norway; 4 Institut Pasteur, Paris, France; University of Wisconsin-Milwaukee, United States of America

## Abstract

PlcR is a *Bacillus cereus* transcriptional regulator, which activates gene expression by binding to a nucleotidic sequence called the ‘PlcR box’. To build a list of all genes included in the PlcR regulon, a consensus sequence was identified by directed mutagenesis. The reference strain ATCC14579 sequenced genome was searched for occurrences of this consensus sequence to produce a virtual regulon. PlcR control of these genes was confirmed by comparing gene expression in the reference strain and its isogenic Δ-*plcR* strain using DNA microarrays, *lacZ* fusions and proteomics methods. The resulting list included 45 genes controlled by 28 PlcR boxes. Forty of the PlcR controlled proteins were exported, of which 22 were secreted in the extracellular medium and 18 were bound or attached to cell wall structures (membrane or peptidoglycan layer). The functions of these proteins were related to food supply (phospholipases, proteases, toxins), cell protection (bacteriocins, toxins, transporters, cell wall biogenesis) and environment-sensing (two-component sensors, chemotaxis proteins, GGDEF family regulators). Four genes coded for cytoplasmic regulators. The PlcR regulon appears to integrate a large range of environmental signals, including food deprivation and self cell-density, and regulate the transcription of genes designed to overcome obstacles that hinder *B. cereus* growth within the host: food supply, host barriers, host immune defenses, and competition with other bacterial species. PlcR appears to be a key component in the efficient adaptation of *B. cereus* to its host environment.

## Introduction

In pathogenic bacteria, the production of virulence factors is often coordinately regulated in response to changes in the bacterial cell environment, with various types of regulatory processes being employed. In Gram-positive bacteria, these processes may involve two-component systems [1], [2], alternative sigma factors [3] or stand-alone transcription regulators [4]. In some cases, the three regulatory mechanisms act together, each controlling a part in the production of virulence factors. This situation is found for instance in the nosocomial infection agent *Staphylococcus aureus*, in which more than 40 cell-surface or secreted proteins involved in bacterial virulence are controlled by a complex network involving the transcriptional regulator SarA, the two component regulator Agr and the general stress response regulator SigB [5]. In some species, a master regulator controls most of the virulence factors, which are therefore members of the same regulon. Virulence regulons may include a large number of genes: for example, the PrfA regulon of the food-borne pathogen *Listeria monocytogenes* includes 73 genes located on the chromosome [6]. Functional analysis of genes included in virulence regulons and a precise understanding of their regulation provide means to determine how environmental signals are integrated by virulence regulators and which strategies are used by bacterial cells to survive and develop within their host environment.

In *Bacillus cereus*, the transcriptional regulator PlcR (Phospholipase C Regulator) controls most known virulence factors [7]. *B. cereus* is a sporulating low-GC Gram-positive bacterium widely distributed in the environment and genetically close to two other pathogens: the human pathogen *B. anthracis*, which is the cause of anthrax and was implicated in the killing of five people in the US in the fall of 2001, and the insect pathogen *B. thuringiensis*. *B. cereus* is a food-poisoning pathogen frequently diagnosed as the causative agent of gastroenteritis [8] but it may also cause more severe diseases such as endophthalmitis [9] or meningitis [10]. PlcR controls the expression of several enterotoxins, haemolysins, phospholipases and proteases [7], [11]. PlcR has been shown to bind to DNA on a specific sequence called the ‘PlcR box’, located upstream from controlled genes, and at various distances ahead of the −35 box of the sigma A promoter [11], [12]. The transcription of *plcR* starts shortly before the onset of the stationary phase t_0_ and reaches a plateau two hours later (t_2_) [13]. *plcR* transcription is autoinduced [13], and is repressed by the sporulation factor Spo0A [14]. PlcR needs PapR to be active: this peptide is expressed as a propeptide under the control of PlcR, is exported out of the cell, is processed to form the active peptide either during export or in the extracellular medium, and is captured back by the cell through the oligopeptide permease system OppABCDF [12], [15], [16]. Thus, the three partners PlcR, OppABCDF and PapR function as a quorum-sensing system. Therefore, PlcR integrates at least two classes of signals: cell growth state through Spo0A and self cell density through PapR [12], [14].

Although several *B. cereus* genes have been demonstrated to be controlled by PlcR, no detailed study of the whole PlcR regulon has been undertaken until now. Moreover, several *B. cereus* group genomes have now been sequenced, presenting the possibility of building a virtual PlcR regulon by searching for matches with the PlcR box consensus sequence. Using this method, a virtual regulon was in fact proposed after the sequences of *B. cereus* ATCC14579 and other strains were published [17]–[19]. However the presence of a PlcR box is not sufficient to classify a gene as PlcR-regulated: experimental evidence is also required. In addition, PlcR may recognize sequences diverging from the previously defined consensus sequence, as has been reported in a study of metalloprotease gene *inhA2* regulation [20]. In order to define all factors involved in the coordinated PlcR-based virulence response in *B. cereus*, we have undertaken an extensive study to map the complete PlcR regulon in the ATCC 14579 reference strain, utilizing mutagenesis experiments and *in silico* predictions in combination with proteomics and transcriptomics analyses.

## Results

### Directed mutagenesis defines a new PlcR box

A consensus sequence was previously determined by alignment of the promoter regions of 13 PlcR-controlled genes [11]. However, the PlcR box located upstream from *inhA2*, a gene known to be under PlcR control, diverged from this original consensus sequence by one base. Therefore, we investigated which substitution could be introduced in the consensus sequence while still maintaining significant PlcR-dependent activity. Thus, we mutated half of the palindromic nucleotide sequence of the PlcR box located upstream from *plcA*, a PlcR-regulated gene coding for a phosphatidyl-inositol specific phospholipase C (PI-PLC) and used to report PlcR activity [12]. The recognition of the mutated PlcR boxes by PlcR was investigated in the ATCC14579 strain by a transcriptional fusion between the modified promoter region of *plcA* and *lacZ*, carried on the pHT304-18Z plasmid [21]. The results, displayed in Table 1, showed that the first and the last nucleotide of the 16 bp consensus sequence can be replaced by any base without a greater than five-fold drop in expression of *plcA-lacZ*. However, deletion of A or T or their replacement by C or G in position 7–8 in the middle of the sequence, or a replacement of C by G in position 5 of the sequence, leads to a dramatic loss of activity. Similarly A_2_, T_3_ or G_4_ could not be replaced by another base. Therefore, the PlcR target sequence identified in the mutagenesis experiment was ‘ATGhAwwwwTdCAT’, were h, w and d stand for, respectively: C, A or T; A or T; G, A or T. In addition to the previous consensus sequence ‘TATGnAnnnnTnCATA’, this sequence was retained for the subsequent *in silico* analysis step.

**Table 1:** Effect of base mutation on the *plcA* PlcR box activity.

	T_1_	A_2_	T_3_	G_4_	C_5_	A_6_	A_7_	T_8_	A_9_	T_10_	T_11_	T_12_	C_13_	A_14_	T_15_	A_16_
A	**20**	**100**	**100**	**1**	*ND*	**100**	**100**	*ND*	*ND*	*ND*	*ND*	*ND*	*ND*	*ND*	*ND*	**100**
T	**100**	*ND*	*ND*	*ND*	*ND*	**4**	**10**	**100**	*ND*	*ND*	*ND*	*ND*	*ND*	*ND*	*ND*	**90**
G	**70**	**1**	*ND*	**100**	**6**	**3**	**3**	**2**	*ND*	*ND*	*ND*	*ND*	*ND*	*ND*	*ND*	**40**
C	**20**	*ND*	**0**	*ND*	**100**	**2**	**2**	**7**	*ND*	*ND*	*ND*	*ND*	*ND*	*ND*	*ND*	**45**
	T	A	T	G	N	A	N	N	N	N	T	N	C	A	T	A

Each column corresponds to each position of the PlcR box located in the region upstream from *plcA* (BC3761). The unmodified sequence is given in the first line, and the subsequent next lines give the effect of a base exchange by A, T, G or C. Last line gives the original consensus sequence. The *plcA* promoter regions including the modified PlcR boxes are transcriptionally fused with *lacZ*, and each modified PlcR box activity is expressed as the percentage of beta-galactosidase activity relative to the unmodified PlcR box. *ND* means ‘not determined’.

### Searching for PlcR boxes in the sequenced *B. cereus* genome

We searched for the two PlcR target sequences TATGnAnnnnTnCATA and ATGhAwwwwTdCAT in the *B. cereus* ATCC14579 genome sequence. We identified a total of 69 boxes located at least 35 bp, but not more than 700 bp, upstream from a putative coding sequence. These boxes may control as many as 138 genes, as the same box could act on several genes putatively organized into an operon and/or that were divergently transcribed (see supplementary material, Table S1). Included in this list are genes that have not been annotated in the published genome sequence of the ATCC14579 strain (Ivanova *et al*., 2003; http://www.ncbi.nlm.nih.gov/genomes/lproks.cgiview1), but for which expression was confirmed by proteomic or genetic means (Bc0361a, this study; BC3763, [7]; Bc2463a, Bc3185a, Bc5101a, [22]).

### Microarray analysis

**Figure 1:**
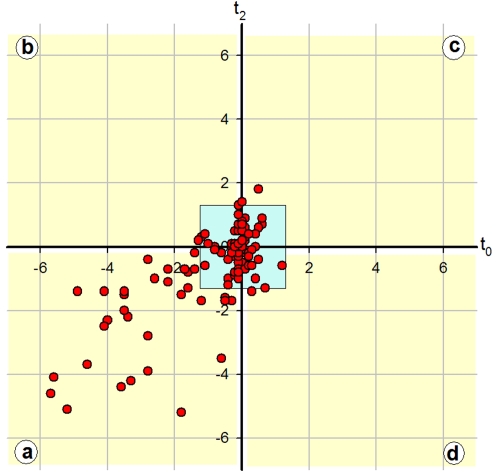
ΔplcR-wt expression ratios as determined by microarray experiments. Ratios of expression between the wildtype strain and the delta *plcR* strain as determined by microarrays. The log2 of these ratios were plotted at t_2_ *vs* t_0_. Each red circle represents the values obtained for one gene. Inside the blue square at the center of the figure are the genes for which the expression ratios were equal or less than 2. Genes for which the transcription was induced by PlcR both at t_0_ and at t_2_ are in the yellow square ‘a’, whereas genes induced only at t_2_ or only at t_0_ are in the yellow squares ‘b’ or ‘d’, respectively. Genes repressed by PlcR both at t_0_ and at t_2_ are in the yellow square ‘c’.

We used microarrays to determine the ratio of expression between the wild type ATCC14579 strain and the isogenic Δ*plcR* strain for the 138 genes identified in the *in silico* procedure. For this determination, we chose two time points in the growth curve: the onset of the stationary phase (t_0_), because PlcR expression increases sharply at this point, and two hours later (t_2_), after PlcR expression reaches a plateau. Most of the genes, which on the basis of genome annotation were expected to be transcribed as part of an operon structure, displayed similar expression ratio values. Only genes with a relative expression ratio greater than 2.5 at t_0_ or at t_2_, and a significance value (p) smaller than 0.2, were considered for subsequent analysis. Consequently, 75 genes (Figure 1, blue box) were considered as not controlled by PlcR under standard culture conditions and were discarded. No microarray data were available for 25 genes. For most of the 38 remaining genes, transcription was enhanced by PlcR both at t_0_ and at t_2_ (Figure 1). Noticeably, the *clO* hemolysin, the *cytK* cytotoxin and the enterotoxins *hblC*, *hbLD*, *hblA* and *nheA*, *nheB*, *nheC*, were the genes most strongly induced by PlcR with relative expression ratios of 10 to 50 in the Δ*plcR* mutant versus wildtype cells. The expression of genes coding for other secreted proteins, including proteases (*sfp*, *nprB*, *nprC*, *mpbE*, *colA*, and *colC*) and phospholipases (*plcA*, *plcB* and *smase*) was also induced by PlcR, with a ratio of induction ranging from 3 to 30 (see supplementary Table S1). The expression of a high number of genes coding for cell-surface proteins appeared also to be induced by PlcR, although at a lower level than for secreted proteins. By contrast, only a few genes coding for cytosolic proteins, including four transcriptional regulators, had their expression significantly induced by PlcR. Data relating to *plcR* and *papR* were discarded, because insertion of the Km^R^ cassette into the *plcR* gene introduced a promoter upstream from the regions recognized by the microarray 70-mer oligos. These genes have however previously been shown to be PlcR-regulated [11], [13]. Finally, 6 genes appeared to be repressed by PlcR at a ratio of 2 to 6 in this microarray analysis, either at t_0_ (BC4986) or at t_2_ (BC0069, BC1736, BC3520, BC4982, BC4983).

### Transcriptional fusions

**Figure 2:**
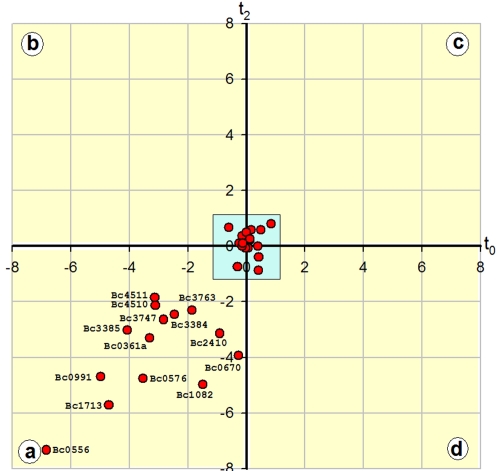
ΔplcR-wt expression ratios as determined by *lacZ* fusions. Ratios of expression between the wildtype strain and the delta *plcR* strain as determined by lacZ fusions. The log2 of these ratios were plotted at t_2_ *vs* t_0_. Each red circle represents the values obtained for one gene. Inside the blue square at the center of the figure are the genes for which the expression ratios were equal or less than 2. The transcription of all the other genes was induced by PlcR both at t_0_ and at t_2_ (yellow square ‘a’).

Results from the microarray analysis were then crossed with data from previous proteomic or genetic analysis [7], [11]–[13], [22]–[24]. All genes encoding secreted proteins and identified by DNA microarray analysis were confirmed as belonging to the PlcR regulon, including BC2463a, BC3185a and BC5101a for which microarray results could not be produced, except for *colC*, for which no data from previous reports was available. Some cell surface and cytosolic proteins (*inhA2*, *prp2* and *plcR*) were also confirmed as part of the PlcR regulon. However, for 39 genes, some of which were in operon, no previous results were available regarding their control by PlcR. Therefore, we constructed 30 transcriptional fusions between the genes promoter region including the PlcR box and a *lacZ* gene, to determine if they were truly controlled by PlcR. Two genes that were not previously predicted to be in the ATCC14579 strain genome sequence, but which were located downstream from PlcR boxes, were included in the analysis: *cwh* (BC3763) and a small open reading frame located downstream from BC0361, which we named BC0361a. The ratios of β-galactosidase (encoded by *lacZ*) activity between the wild type strain and the *ΔplcR* strain was plotted at t_0_ *vs* t_2_ (Figure 2; the kinetics of expression obtained between t_−1_ and t_4_ are shown in supplementary, Figure S1). Among the 30 promoter regions assayed by *lacZ* fusions, 14 were controlled by PlcR whereas the remaining 16 were not. Microarray results were missing or gave low ratio values for these 16 PlcR-independent genes.

### Proteomic study

We have observed in a previous two-dimensional protein gel electrophoresis analysis of the *B. cereus* ATCC14579 secretome at t_2_ that most of the extracellular proteins disappeared upon inactivation of *plcR* [7]. However, in the same time a large number of spots appeared on the gel obtained from the mutant strain. This suggests that PlcR is possibly a repressor for some extracellular proteins. We identified the protein content of 103 of these spots by peptide mass fingerprints and by N-terminal sequencing (see supplementary material, Figure S2 and Table S2), and only one of the proteins was encoded by a gene preceded by a PlcR box. This protein, the fructose bisphosphate aldolase FbaA, was shown by *lacZ* fusion not to be controlled by PlcR (Figure S1). Therefore no repressor role for PlcR acting on genes coding for secreted proteins was identified. The appearance of cytosolic proteins in the culture supernatant if *plcR* is inactivated was due to a greater cell lysis in the mutant strain than in wildtype: this lysis, determined by measuring isocitrate dehydrogenase activity in the bacterial cells and culture supernatant, was 1% in the wildtype strain and 15% in the mutant strain at t_2_.

### Final list of the PlcR-controlled genes

We built a list of PlcR-controlled genes using the data generated. We added three genes coding for antibacterial peptides to this list, which were previously shown to be controlled by PlcR: *sppc1*, *sppc2* and *sppc3* [22]. The final list included 45 genes controlled by 28 PlcR boxes, as the same PlcR box may control several genes (Table 2 and Figure S4). Genes coding for secreted proteins made up 49% of the regulon, whereas genes coding for proteins associated to the membrane or to the peptidoglycan (cell wall proteins) represented 40%. The 22 secreted proteins were toxins, phospholipases, proteases, peptides with antibacterial activity and included one cell-cell communication peptide (PapR); however, the 18 cell wall proteins were annotated as being involved in cell immunity, drug efflux transport, cell wall biogenesis, and environment-sensing (in connection with regulation systems). Environmental sensors included two chemotaxis proteins, McpA and TlpA, the two-component system sensor YufM and one GGDEF family protein. The GGDEF family protein displays three conserved domains: a dinucleotide cyclase and a phosphodiesterase domain, involved in regulating the intracellular level of cyclic dinucleotide diguanylate, a second messenger, in response to ligands detected by the PAS domain [25]. One protein, InhA2, was possibly involved in cell immunity. InhA2 is a member of the Immune Inhibitor A metalloprotease family, previously shown to specifically degrade antibacterial peptides [26] and involved in bacterial virulence [20]. The cytosolic proteins controlled by PlcR were PlcR itself, a TetR family regulator, a two-component response regulator, a protein of unknown function and a protein homologous to the RimL ribosomal alanine acetyl transferase. Therefore, all cytoplasmic PlcR-controlled proteins of known function are likely to be regulators. The TetR family regulators are transcriptional repressors involved in the biosynthesis of antibiotic efflux pumps and the response to osmotic stress [27]. The two-component response regulator YufM is in an operon with its sensor component YufL and a chemotaxis transducer protein McpA, all under PlcR transcriptional control. RimL belongs to the GNAT superfamily of acetyltransferases [28] and acetylates a ribosomal protein interacting with elongation factors EF-Tu and EF-G [29], [30].

**Table 2:** List of the PlcR-controlled genes in the ATCC14579 strain.

Gene n°	Gene ID	Name	Function	Localisation
Bc1809	30019951	*nheA*	Enterotoxin	Extracellular
Bc1810	30019952	*nheB*	Enterotoxin	Extracellular
Bc1811	30019953	*nheC*	Enterotoxin	Extracellular
Bc3102	30021214	*hblB*	Enterotoxin	Extracellular
Bc3103	30021215	*hblL1*	Enterotoxin	Extracellular
Bc3104	30021216	*hblL2*	Enterotoxin	Extracellular
Bc5101	30023138	*clo*	Hemolysin I, cereolysin	Extracellular
Bc1110	30019265	*cytK*	Hemolysin, cytotoxin	Extracellular
Bc3761	30021854	*plcA*	Phospholipase (phosphatidyl inositol)	Extracellular
Bc0670	30018852	*plcB*	Phospholipase (phosphatidyl choline)	Extracellular
Bc0671	30018853	*smase*	Phospholipase (sphingomyelin)	Extracellular
Bc2735	30020906	*nprP2*	Neutral protease	Extracellular
Bc3383	30021487	*nprC*	Neutral protease	Extracellular
Bc5351	30023381	*nprB*	Neutral protease	Extracellular
Bc0556	30018742	*colC*	Protease, collagenase	Extracellular
Bc3161	30021271	*colA*	Protease, collagenase	Extracellular
Bc3384	30021488	*mpbE*	Protease, Enhancin	Extracellular
Bc3762	30021855	*sfp*	Protease, subtilase family protease	Extracellular
Bc5101a	NA	*sppc1*	Peptide with anti-bacterial activity	Extracellular
Bc2463a	NA	*sppc2*	Peptide with anti-bacterial activity	Extracellular
Bc3185a	NA	*sppc3*	Peptide with anti-bacterial activity	Extracellular
Bc5349	30023379	*papR*	Peptide, signaling molecule	Extracellular
Bc0576	30018762	*mcpA*	Methyl-accepting chemotaxis transducer protein	Cell wall
Bc3385	30021489	*tlpA*	Methyl-accepting chemotaxis transducer protein	Cell wall
Bc0577	30018763	*yufL*	Two-component system sensor	Cell wall
Bc3747	30021841		sensory box / GGDEF family protein	Cell wall
Bc4509	30022587		ABC transporter, permease subunit	Cell wall
Bc4510	30022588		ABC transporter, ATP-binding protein	Cell wall
Bc2411	30020542		Drug efflux protein	Cell wall
Bc3763	NA	*cwh*	Cell wall hydrolase	Cell wall
Bc0991	30019146	*slpA*	S-layer protein A, autolysin	Cell wall
Bc3746	30021840		Predicted hydrolase or acyl transferase. Lipoprotein?	Cell wall
Bc0666	30018848	*inhA2*	Metalloprotease – lipoprotein	Cell wall
Bc4999	30023039		CAAX amino terminal protease family, 6 TM domains	Cell wall
Bc4511	30022589	*lppC*	Acid phosphatase, lipoprotein	Cell wall
Bc2552	30020679		Unknown, 2 transmembrane domains	Cell wall
Bc1713	30019857		Unknown, membrane spanning protein	Cell wall
Bc3527	30021629		Unknown, membrane spanning protein	Cell wall
Bc0361a	NA		Unknown, 1 TM domain	Cell wall
Bc0362	30018570		Unknown, lipoprotein	Cell wall
Bc0578	30018764	*yufM*	Two-component system regulator	Cytoplasm
Bc2410	30020541	*tetR*	Regulator, TetR family	Cytoplasm
Bc1082	30019237		Ribosomal protein alanine acetyl transferase; regulator ?	Cytoplasm
Bc5350	30023380	*plcR*	Transcriptional regulator	Cytoplasm
Bc1081	30019236	*prp2*	Unknown	Cytoplasm

Sppc stands for ‘small peptide regulated by PlcR in *B. cereus*’. Sppc genes are wrongly annotated in the ATCC14579 genome. Bc2463a, BC3185a and Bc5101a are located between the PlcR box and, respectively, Bc2463, Bc3185 and Bc5101. Bc0361a is located between the PlcR box and Bc0361. Overall, 22 PlcR-controlled proteins are secreted, 18 are located in the cell wall and 5 are located in the cytosol. Determination of protein subcellular localisation was based on signal peptides, hydrophobic domains and cell-wall/membrane anchoring motifs presence.

### Analysis of the nucleotidic sequences of the active PlcR boxes

**Figure 3:**
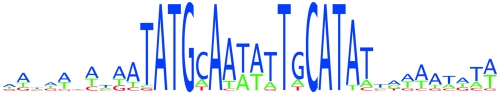
PlcR consensus sequence. The height of the letter representing a base is proportional to its frequency at each position in the alignment. For each position, the most frequent base is drawn in blue, followed by green and pink for less frequent bases.

**Figure 4:**
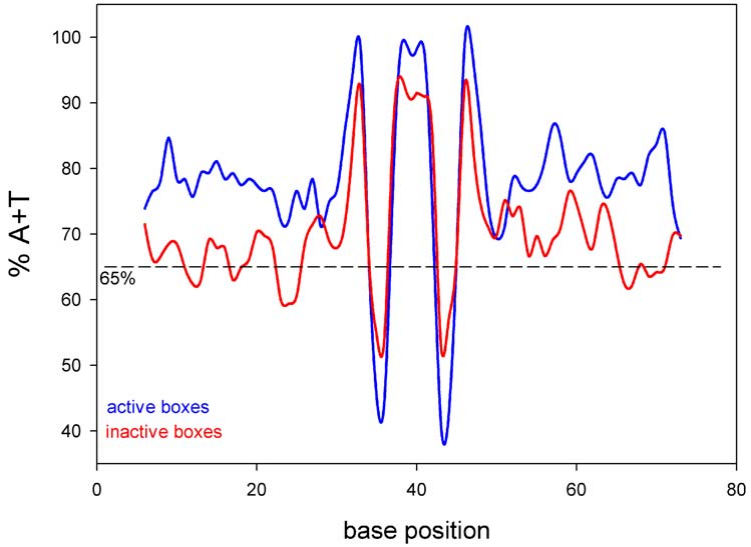
Percentage of A+T in the vicinity of PlcR boxes. Active boxes are plotted in blue whereas inactive boxes are plotted in red. The dashed line represents the average A+T percentage for the ATCC14579 chromosome. The difference between active and inactive boxes for the A+T percentage is highly significant (Qui-square test, p<0.001).

The 28 active PlcR boxes that we determined were scattered all along the chromosome (Figure S3, supplementary material). Consequently, no pathogenicity island could be found in the *B. cereus* chromosome. Alignment of the active PlcR boxes led us to a new consensus sequence, shown as a logo in Figure 3. To investigate whether nucleotide sequences surrounding active PlcR boxes could exhibit additional properties required for the box to be recognized by PlcR, a comparison of sequences upstream or downstream from the active and inactive PlcR boxes was performed. We found that in the vicinity of the active PlcR boxes, the AT-content was much higher than in the vicinity of inactive boxes (Figure 4). Downstream from all the active PlcR boxes, we identified a putative −10 σ^A^ binding sequence (Figure S5), suggesting that the PlcR-regulated genes may be transcribed by a σ^A^-associated RNA polymerase.

## Discussion

### Building a list of PlcR-controlled genes

In 1999, Agaisse and colleagues used a genetic screen to identify PlcR-regulated genes. They reported 13 genes encoding exported proteins, mostly toxins and degradative enzymes [11]. As a consequence, PlcR appeared to be a pleiotropic virulence regulator controlling extracellular factors. That study also led to the definition of a PlcR target sequence, to which the active complex PlcR/PapR binds [12]. Later, the sequencing of *B. cereus* genomes in combination with proteomic and genetic studies revealed that PlcR may control a much higher number of genes, all of which were not, at least not directly, involved in virulence [7], [17], [24], [31]. Indeed, the role of PlcR in virulence has been extensively documented [31]–[34]. Various studies have also suggested that PlcR could acts on other functions, including sporulation [35] and biofilm formation [36]. We therefore systematically investigated the PlcR regulon to understand better the role of PlcR during bacterial infection, and have provided the first comprehensive, genome-wide characterization of the complete PlcR virulence regulon based on functional experiments. A virtual PlcR regulon was constructed *in silico* using the PlcR DNA target sequence defined through mutagenesis experiments, and was investigated by transcriptional studies using DNA microarrays and *lacZ* fusions. The resulting data were cross-analyzed with data from proteomic studies, to build a list of 45 genes positively controlled by PlcR under standard culture conditions. The genes were scattered along the chromosome, and did not form a pathogenicity island. Aligning the sequences of the PlcR boxes located upstream from these genes led to the identification of the PlcR consensus binding sequence wTATGnAwwwwTnCATAw.

### Inactive PlcR boxes

A high number of PlcR boxes turned out to be inactive under our culture conditions. Alignment of the sequences located upstream and downstream from the PlcR boxes revealed that, for active boxes, these sequences are significantly more AT-rich. Therefore, in addition to the consensus sequence, the genetic environment of the PlcR box could be critical for the binding of PlcR to its box, and/or could be important for the transcription activity of the promoter. Also, we found cases in which a PlcR box is placed between two divergently transcribed genes, and PlcR controls the transcription of only one of these genes (for example, Bc0555/Bc0556). Thus, the binding of PlcR to its box is required but not sufficient to activate the transcription of genes located downstream. A putative σ^A^ -10 region consensus sequence was found downstream from all the active PlcR boxes. In various conditions, including the host, the transcription of genes not controlled by PlcR despite the binding of the regulator to the PlcR box may require alternative sigma factors, such as σ^B^, σ^H^, or ECF factors. In *B. anthracis* [37], *L. monocytogenes* [38], [39] and *S. aureus* [5], σ^B^ has been shown to be involved in virulence under some culture conditions. Similarly, σ^H^ is required for toxin gene expression in *B. anthracis* [40]. These two sigma factors are likely to be expressed in early stationary phase under standard culture conditions [37], [40]. Finally, it seems unlikely that we may have missed any PlcR-regulated genes in this study due to no expression in LB medium because no additional genes were identified in a screen for genes specifically induced during growth *in vivo* [31].

### Role of the PlcR regulon

Ninety percent of the genes included in the final list of PlcR-controlled genes encode proteins either secreted or located at the cell wall, i.e. at the interface between the bacterial cell and its environment – including the eukaryotic host. Proteases and phospholipases, in addition to enterotoxins and hemolysins, have been found located at this interface. These enzymes are likely to be involved in host tissue degradation. Phosphatidylcholine-specific phospholipase C (PC-PLC) and sphingomyelinase were previously shown to induce hemolysis [41], [42] and the InhA2 metalloprotease is involved in protecting the bacterial cell from host immune defenses [26]. Proteins involved in peptidoglycan synthesis and modification (four genes) are also likely to be involved in bacterial cell protection by strengthening the cell wall, as suggested by the significantly greater tendency of cell lysis observed for the *plcR* mutant strain than the wildtype strain (this study). Furthermore, three secreted antibacterial peptides and four drug efflux transporters shown to be controlled by PlcR for the first time here may protect the cell from competition with other bacterial species and their bacteriocins.

**Figure 5:**
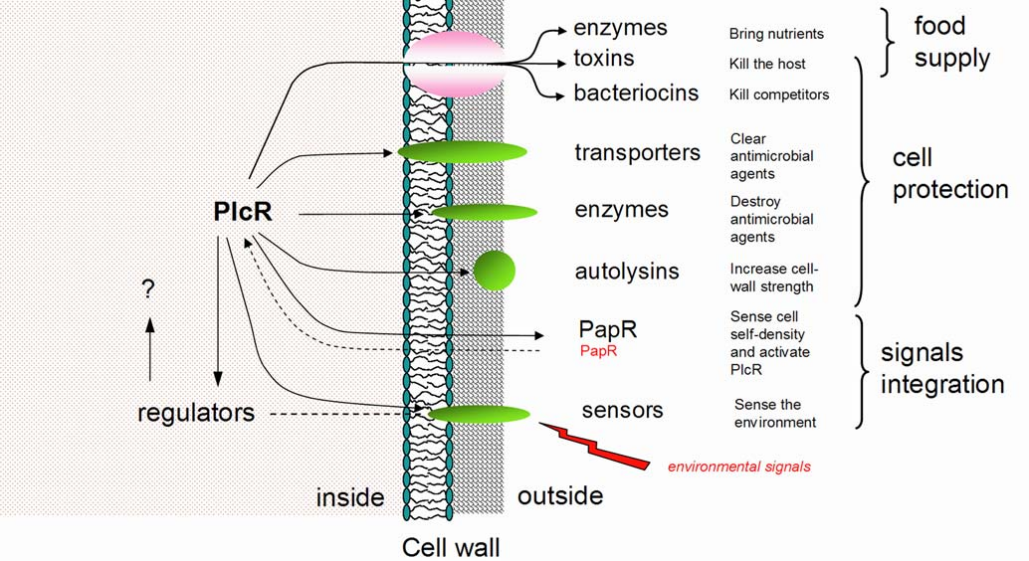
Overview of the PlcR regulon organization. PlcR positively controls (dark line) the transcription of a vast array of genes coding for proteins located in the cell wall or in the extracellular space. Cell wall proteins are designed in green. Secreted proteins are exported through the SecA machinery designed in pink. Environmental signals are sensed by cell-wall sensors and act *via* (dashed line) regulators on undetermined genes or proteins. PlcR requires PapR to be active (dashed line). Signals integrated by PlcR and PlcR-controlled regulators are designed in red.

Thus, these functions may work together to provide nutrition and bacterial cell protection in a hostile host environment (Figure 5). The bacterium may feed on host tissues by producing toxins, phospholipases and proteases. Proteins, peptides and amino acids have been suggested as the preferred nutrient sources for *B. cereus* [17], possibly linked to the growth of the bacterium as a human and animal pathogen. Meanwhile, other functions of the regulon may inhibit the growth of other bacterial species in the same niche, inactivate host antibacterial peptides, and increase cell wall resistance to lysis.

### Sensing the host environment

Sensing the surrounding environment is necessary for a bacterium to react appropriately to changes. Bacterial pathogens often use two-component systems to sense their host environment, and promote or repress the transcription of genes in response to changes in this environment [1], [2]. Interestingly, as shown here for the first time, four sensors are included in the *B. cereus* PlcR virulence regulon, only one of which (YufL) is part of a two-component system. The other sensors are chemotaxis proteins (McpA and TlpA) or a GGDEF-family regulator producing a second messenger. This variety in sensor types is likely to reflect a variety in the types of signals providing input to the cell. Furthermore, genes controlled by PlcR-dependent transcriptional regulators could add to the list of genes controlled by PlcR, and extend the regulon size. However, these regulators could also recruit genes already controlled by PlcR. If so, PlcR-controlled sensors and their regulators could modulate the transcription of subgroups of PlcR-controlled genes. It was recently suggested that regulators other than PlcR could act on the expression of PlcR-controlled genes [43], [44]. Accordingly, *L. monocytogenes* internalins, which constitute a subgroup of the PrfA virulence regulon, are simultaneously controlled by σ^B^, itself acting on gene transcription in response to stress signals [39]. These observations have led us to propose the following hypothesis for the integration of PlcR/PapR-related environmental signals in *B. cereus*: PlcR triggers the transcription of its regulon, including the sensor proteins, in response to food deprivation sensed *via* transition state regulators and in response to its own cell density sensed *via* PapR. In turn, the PlcR-controlled sensors repress (or promote) transcription of subgroups of genes in response to host signals. The PlcR/PapR quorum-sensing system thus provides an efficient way to integrate several environmental signals and produce a gene expression profile continuously adapted to a changing host environment, such as that experienced by the bacterium during infection.

## Materials and Methods

### Strains and culture conditions

The *B. cereus* strains used in this study were the type strain, ATCC14579, and the isogenic Δ*plcR* strain [32], obtained by insertion of a Km^R^ cassette in *plcR*. The two strains were grown in Luria Bertani broth (LB) at 30°C. Cultures were harvested at the onset of the stationary phase (t_0_) or two hours later (t_2_). The onset of the stationary phase (t_0_) was defined as the breakpoint in the vegetative phase slope.

### Directed mutagenesis of the PlcR box

Point mutations were introduced into the PlcR box of the *plcA* promoter region by PCR amplification with primer Bc-plc matching the 5′ end of the *plcA* gene, and primers pRX1 to pRX18 carrying a modified PlcR box (supplementary Table S3). Each PCR product (a 390-bp DNA fragment) was digested with *Xba*I and *Hin*dIII enzymes and cloned between the *Xba*I and *Hin*dIII sites in pHT304-18Z [21]. The nucleotide sequence of each DNA fragment was determined and analyzed by Genome express (France) by using oligonucleotides UP and OVG flanking the DNA fragments cloned into pHT304-18Z. Plasmids carrying the *plcA'-lacZ* transcriptional fusions were introduced into *B. cereus* ATCC14579 by electroporation, and ß-galactosidase activity produced by the recombinant clones was measured two hours after entry in the stationary phase in LB medium.

### Microarray analysis

Harvested bacterial cells were incubated in an equal volume of ice-cold methanol for 5 minutes before centrifugation at 4°C and 4000 rpm. RNA isolation was performed with the RNeasy Midi Kit (Qiagen, Germany) together with the RNase-Free DNase Set (Qiagen, Germany). For microarray slide preparation, 70-mer oligos from the whole genomic *B. cereus* ATCC14579 ORFs (released at NCBI in 2003) were designed and synthesized by Qiagen-Operon (Germany). The oligos were printed in 50% DMSO on UltraGAPS™ gamma amino silane-coated slides from Corning (USA), at the Norwegian Radiumhospital (DNR). The microarray slides were prehybridized before use for 30–60 minutes in a 5×SSC/0.1 % SDS/0.1 % BSA solution at 42°C, according to the UltraGAPS™ Coated Slides instruction manual from Corning. The slides were then washed three times in MQ H_2_O, once in isopropanol and finally spun dry.

cDNA synthesis, labeling and purification was carried out with the FairPlay™ microarray labeling kit (Stratagene, CA, USA), using 500 ng random hexamers (Applied Biosystems, CA, USA) on 20 μg of RNA, and with amino-allyl coupling of Cy3 and Cy5 dyes from Amersham Biosciences (GE Healthcare Bio-Sciences AB, Sweden). After purification, the samples were concentrated with a Microcon column (Millipore, MA, USA) and hybridization solution was added to a final concentration of 30 % formamide, 5×SSC, 0.1 % SDS and 0.1 mg/mL sperm DNA, based on the UltraGAPS™ Coated Slides instruction manual from Corning (USA). Labeled DNA were denatured at 95°C for 2 minutes, and incubated at 42°C before hybridization. The samples were hybridized in a hybridization chamber (Monterey Industries, CA, USA), humidified with 5×SSC for 16 hours in a 42°C water bath. After hybridization, the slides were washed at 42°C in 0.5×SSC/0.01 % SDS and in 0.06×SSC, and finally at room temperature in isopropanol before they were spun dry.

The slides were scanned with an Axon 4000B scanner. Gridding, spot annotation and calculation was carried out using GenePix Pro 6.0 software. The R platform [45] and LIMMA [46], [47] were used for filtering, normalization and further analysis (for details, see supplementary material). P-values were computed using false discovery rate correction of 0.05.

### Transcriptional fusions

Transcriptional fusions were constructed in the pHT304-18Z plasmid, between the X*ba*I and P*st*I or H*in*dIII and B*am*HI cloning sites of the plasmid [48]. Primers used for PCR-amplification of the promoter regions cloned are listed in supplementary Table S4. The resulting plasmids were then transferred into *B. cereus* strains ATCC14579 or ATCC14579 Δ*plcR* by electroporation. For β-galactosidase activity measurement, bacterial cells were lysed using the FastPrep 120 system (Savant), and β-galactosidase-specific activities were measured as described previously [49]. The specific activities are expressed in units of β-galactosidase milligram^−1^ of protein (Miller units). Two to four assays were performed for each transcriptional fusion.

### Two-dimensional electrophoresis

Protein extracts were prepared from the culture supernatants and subjected to two-dimensional electrophoresis as described earlier [7]. The spots were immediately excised and stored at −70°C until use. Proteins were identified by peptide mass fingerprint and by N-terminal sequencing. Peptide mass fingerprints were generated after trypsin-digestion and MALDI-TOF analysis (Biobac, INRA, Jouy-en-Josas, France), and proteins were identified using ProteinProspector or Mascot programs. N-terminal sequencing was performed by Prof. K. Sletten at the Biotechnology Center, University of Oslo, Norway.

### Sequence analysis

PlcR boxes were searched in the sequenced genome of the ATCC14579 strain using the ‘find sequence’ tool of Vector NTI (Invitrogen). The same method was used to find σ^A^ −10 boxes in the promoter regions of PlcR-controlled genes. The consensus sequence for PlcR binding (‘PlcR box’) was drawn as a logo where, at each nucleotide position, the letter height is proportional to the frequency of the base and to the weight of the position in the sequence [50]. The content in A+T bases in sequences upstream and downstream from the inactive and active PlcR boxes were compared using a chi-square test.

## Supporting Information

Table S1 Microarray results for genes with a PlcR box in their promoter region
(0.26 MB PDF)

Table S2 Proteins identified in the culture supernatant of the delta-PlcR ATCC14579 strain harvested at t2
(0.19 MB PDF)

Table S3 Primers used for the directed mutagenesis of the PlcR box
(0.04 MB PDF)

Table S4 Primers for transcriptional fusions
(0.05 MB PDF)

Figure S1 Results from lacZ fusions
(0.13 MB PDF)

Figure S2 Two-dimensional gel electrophoresis of the Δ-plcR ATCC14579 supernatant
(0.97 MB PDF)

Figure S3 Location of PlcR boxes on the ATCC14579 chromosome
(0.02 MB PDF)

Figure S4 Genetic environment of the 45 PlcR-regulated genes
(0.26 MB PDF)

Figure S5 Putative −10 σ^A^ boxes located downstream of PlcR boxes for PlcR-controlled genes
(0.60 MB PDF)
