# Supplementary material for: The PlcR Virulence Regulon of Bacillus cereus
Source: PLoS One. 2008 Jul 30;3(7):e2793. doi: 10.1371/journal.pone.0002793 (PMC2464732; doi:10.1371/journal.pone.0002793)
Supplement: Table S1 — Microarray results for genes with a PlcR box in their promoter region (0.26 MB PDF) [file pone.0002793.s001.pdf]

gene	name	Function	PlcR box	Location[a]	Dist[b]	r0[c]	p0[c]	r2[c]	p2[c]
BC0069	*spoIIE*	stage II sporulation protein E	catgcaaatttgcatg	64359	257	NA	NA	1.3	0.175
BC0313		Methyltransferase	aatgaaattttgcatt	283956	152	-0.3	0.978	0.0	0.985
BC0360	*ampS*	aminopeptidase AmpS	tatgcaattttgcata	341478	427	0.0	0.998	-0.5	0.005
BC0361		polysaccharide deacetylase-like protein	tatgcaattttgcata	341478	427	-0.6	0.006	-0.2	0.700
BC0361a		unannotated 57-amino acids peptide	tatgcaattttgcata	341478	427	NA	NA	NA	NA
BC0362		hypothetical protein	tatgcaattttgcata	341478	98	-2.8	0.002	-0.4	0.290
BC0442		tellurium resistance protein. putative	tatgaatgttttcata	430642	112	0.6	0.015	0.7	0.000
BC0443		tellurium resistance protein	tatgaatgttttcata	430642	112	0.6	0.032	0.9	0.003
BC0444		tellurium resistance protein	tatgaatgttttcata	430642	112	0.5	0.049	0.6	0.014
BC0445		tellurium resistance protein. putative	tatgaatgttttcata	430642	112	0.1	0.739	0.6	0.104
BC0446		conserved hypothetical protein	tatgaatgttttcata	430642	112	0.4	0.077	0.0	0.970
BC0447		tellurite resistance protein. putative	tatgaatgttttcata	430642	112	-0.1	0.720	0.7	0.043
BC0555		glycine betaine transporter	tatgaaatattgcatt	535372	165	0.1	0.541	-0.2	0.659
BC0556	*colC*	microbial collagenase.putative	tatgaaatattgcatt	535372	94	-3.4	0.000	-2.2	0.002
BC0576	*mcpA*	methyl-accepting chemotaxis protein	tatgcaaaaatgcata	559643	98	-1.6	0.000	-0.8	0.119
BC0577	*yufL*	sensory box histidine kinase	tatgcaaaaatgcata	559643	98	-0.8	0.009	0.0	0.982
BC0578	*yufM*	response regulator	tatgcaaaaatgcata	559643	98	-0.5	0.059	-1.6	0.160
BC0597		nicotinate phosphoribosyltransferase. putative	aatgaaattatgcata	589180	94	-0.2	0.442	-0.3	0.116
BC0598	*nprR*	Transcriptional activator NprR	aatgaaattatgcata	589180	91	NA	NA	0.1	0.934
BC0599		Transposase	aatgaaattatgcata	589180	91	0.5	0.000	-0.4	0.000
BC0600		Transposase	aatgaaattatgcata	589180	91	0.5	0.000	-0.4	0.000
BC0666	*inhA2*	immune inhibitor A metalloprotease	catgcaattttgcata	662055	307	-1.8	0.020	-5.2	0.000
BC0670	*plcB*	phospholipase C	tatgaacatttgcata	668446	124	-4.9	0.001	-1.4	0.007
BC0671	*Smase*	sphingomyelinase C	tatgaacatttgcata	668446	124	-4.1	0.002	-1.4	0.087
BC0672		hypothetical protein	tatgaacatttgcata	668446	124	-2.2	0.025	-0.7	0.156
BC0673		Flavin-dependent dehydrogenase	tatgaacatttgcata	668446	124	-0.3	0.514	-1.7	0.157
BC0757		major facilitator family transporter	catgaaaaattgcata	746136	480	-0.2	0.779	-0.1	0.579
BC0759		phospholipase. putative	catgaaaaattgcata	746136	235	-0.1	0.402	-0.5	0.206
BC0860		Multidrug resistance protein B	tatgaaaatatacatc	834200	682	0.2	0.438	-0.2	0.925
BC0917		hypothetical Cytosolic Protein	tatgtaaaaatgcata	905398	343	-0.2	0.001	0.0	0.895
BC0990		hypothetical protein	tatgaatttttgcata	976350	347	0.1	0.438	-0.1	0.701
BC0991	*slpA*	S-layer protein. putative	tatgaatttttgcata	976350	116	-2.8	0.000	-2.8	0.000
BC0992		hypothetical protein	tatgaatttttgcata	976350	116	-0.2	0.990	0.0	0.994
BC1081	*prp2*	PlcR-regulated protein PRP2	tatgtaatattgcata	1069156	61	-1.1	0.022	-0.6	0.131

gene	name	Function	PlcR box	Location[a]	Dist[b]	r0[c]	p0[c]	r2[c]	p2[c]
BC1082		acetyltransferase. GNAT family	tatgtaatattgcata	1069156	58	NA	NA	NA	NA
BC1110	*cytK*	Cytotoxin K,	tatgcataattgcata	1092541	82	-4.0	0.000	-2.3	0.009
BC1111		phosphoesterase	tatgcataattgcata	1092541	195	0.2	0.387	-0.6	0.055
BC1112		HD domain protein	tatgcataattgcata	1092541	195	-0.4	0.018	-0.4	0.626
BC1349		acetyltransferase. GNAT family	tatggaactttgcata	1323471	42	-0.1	0.561	-0.3	0.119
BC1350		hypothetical protein	tatggaactttgcata	1323471	278	0.1	0.095	0.2	0.373
BC1429		hypothetical protein	tatggaaatttccata	1389495	231	-0,1	0.740	0.1	0.897
BC1641	*flgB*	flagellar basal-body rod protein FlgB	tatgtaagggtacata	1596806	97	-0.4	0.065	-1.2	0.000
BC1642	*flgC*	flagellar basal-body rod protein FlgC	tatgtaagggtacata	1596806	97	-0.4	0.049	-1.0	0.000
BC1643	*fliE*	flagellar hook-basal body complex protein FliE. putative	tatgtaagggtacata	1596806	97	-0.2	0.333	-0.9	0.042
BC1644	*fliF*	Flagellar M-ring protein FliF	tatgtaagggtacata	1596806	97	-0.2	0.133	-0.8	0.027
BC1645	*fliG*	flagellar motor switch protein FliG	tatgtaagggtacata	1596806	97	-0.1	0.508	-0.2	0.646
BC1646		conserved hypothetical protein	tatgtaagggtacata	1596806	97	-0.1	0.742	-0.8	0.168
BC1647		flagellum-specific ATP synthase. putative	tatgtaagggtacata	1596806	97	-0.1	0.615	-0.7	0.146
BC1655		conserved hypothetical protein	tatgcaattttacata	1608869	162	0.4	0.065	0.4	0.346
BC1713		hypothetical Membrane Spanning Protein	tatgcagaattgcata	1662411	68	-2.6	0.000	-1.0	0.000
BC1734		ABC transporter. ATP-binding protein	tatgaaattattcata	1678356	472	0.3	0.409	-0.1	0.864
BC1735		Export ABC transporter permease protein	tatgaaattattcata	1678356	472	0.1	0.679	0.9	0.000
BC1736		Export ABC transporter permease protein	tatgaaattattcata	1678356	472	-0.1	0.854	1.3	0.000
BC1809	*nheA*	Enterotoxin,	tatgcataattgcata	1764706	527	-3.6	0.003	-4.4	0.000
BC1810	*nheB*	Enterotoxin,	tatgcataattgcata	1764706	527	-3.3	0.001	-4.2	0.000
BC1811	*nheC*	Non-expressed Enterotoxin C,	tatgcataattgcata	1764706	527	-4.6	0.000	-3.7	0.000
BC1956		DNA-binding response regulator	gatgcatttattcatg	1903735	457	-0.4	0.073	NA	NA
BC1957		sensor histidine kinase	gatgcatttattcatg	1903735	457	0.7	0.958	-1.3	0.826
BC1975		hypothetical Exported Protein	tatgtaggcatgcata	1922975	458	-0.4	0.079	-1.2	0.000
BC2138		hypothetical protein	aatgaaataatacata	2083572	270	0.1	0.531	0.3	0.378
BC2356		acetyltransferase. GNAT family	gatgaatatatacata	2302720	308	0.1	0.068	-0.7	0.004
BC2409		hypothetical protein	tatgcaatattacata	2353229	74	-1.0	0.141	0.1	0.660
BC2410	*tetR*	Transcriptional regulator. TetR family	tatgcaatattacata	2353229	253	-0.3	0.998	NA	NA
BC2411		ABC transporter. permease protein. putative	tatgcaatattacata	2353229	253	NA	NA	-1.3	0.830
BC2463a	*sppc2*	small peptide PlcR-controlled	tatgcaatattgcata	2427276	90	NA	NA	NA	NA
BC2466	*colB*	Microbial collagenase	tatgtaattttgcata	2431934	525	0.2	0.481	NA	NA
BC2552		hypothetical protein	tatgcaaatatgcata	2526975	82	-4.1	0.000	-2.5	0.000
BC2706		Acetyltransferase	tatgtagctattcata	2687769	299	0.1	0.998	NA	NA

gene	name	Function	PlcR box	Location[a]	Dist[b]	r0[c]	p0[c]	r2[c]	p2[c]
BC2707		Acetyltransferase	tatgtagctattcata	2687769	299	NA	NA	NA	NA
BC2735	*nprP2*	neutral protease	tatgcaaaattacata	2714407	98	-0.6	0.310	-3.5	0.002
BC2767		hypothetical Membrane Associated Protein	tatgtatttatacata	2745399	664	-0.1	0.998	0.0	0.855
BC2768		hypothetical protein	tatgtatttatacata	2745399	664	0.2	0.452	-0.6	0.104
BC2769		acetyltransferase. GNAT family	tatgtatttatacata	2745399	664	NA	NA	-0.8	0.009
BC2770		transcriptional regulator. DeoR family	tatgtatttatacata	2745399	664	NA	NA	NA	NA
BC2798		chitin binding protein. putative	aatgtataatttcatt	2766532	423	-0.1	0.914	-1.0	0.316
BC2922		hypothetical protein	aatgaattaattcatt	2879356	81	-1.2	0.002	0.3	0.836
BC2923		Acetyltransferase	aatgaattaattcatt	2879356	81	-0.3	0.998	NA	NA
BC2924		Acetyltransferase	aatgaattaattcatt	2879356	81	NA	NA	NA	NA
BC3019		hypothetical protein	catgtattaattcatt	2982086	430	-0.1	0.998	-0.8	0.214
BC3102	*hblA*	Hemolysin BL binding component precursor,	tatgtataattgcata	3067278	879	-3.5	0.000	-2.0	0.003
BC3103	*hblD*	Hemolysin BL lytic component L1,	tatgtataattgcata	3067278	879	-5.2	0.000	-5.1	0.000
BC3104	*hblB*	Hemolysin BL lytic component L2,	tatgtataattgcata	3067278	879	-5.6	0.000	-4.1	0.000
BC3161	*colA*	microbial collagenase. putative	tatgtattatttcata	3131867	66	-0.5	0.053	-1.7	0.078
BC3185a	*Sppc3*	small peptide PlcR-controlled	tatgcaatattgcata	3156120	92	NA	NA	NA	NA
BC3383	*nprC*	neutral protease	tatgtaattttgcata	3349558	174	-1.6	0.033	-1.3	0.533
BC3384	*mpbE*	enhancin family protein	tatgtaattttgcata	3349558	174	-1.4	0.075	-0.7	0.309
BC3385	*tlpA*	Methyl-accepting chemotaxis protein	tatgcaattttgcata	3351516	82	-1.2	0.195	-1.7	0.099
BC3461		conserved hypothetical protein	aatgaatatatacatc	3417962	291	-0.2	0.344	0.5	0.111
BC3463		arsenical pump family protein	aatgaatatatacatc	3417962	322	-0.3	0.147	0.1	0.068
BC3520		methyl-accepting chemotaxis protein	tatgcaatattacatg	3479263	616	0.5	0.416	1.8	0.754
BC3521		hypothetical protein	aatgtaatattacata	3481861	560	NA	NA	-0.4	0.297
BC3526		Collagen adhesion protein	tatgcaatattgcatt	3491783	481	0.2	0.419	-0.2	0.777
BC3527		hypothetical protein	tatgcaatattgcatt	3491783	76	-1.7	0.001	-0.7	0.078
BC3528		Sporulation kinase	tatgtaataattcata	3493194	554	0.3	0.409	-0.1	0.539
BC3688		conserved hypothetical protein	aatgcaataattcatc	3656825	615	0.0	0.968	NA	NA
BC3690		LexA repressor	aatgcaataattcatc	3656825	202	0.0	0.885	0.0	0.894
BC3739		exodeoxyribonuclease III	tatgaatacatacata	3704404	96	-0.3	0.010	-0.2	0.156
BC3740		Ada regulatory protein/6-O-methylguanine-DNA methyltransferase	tatgaatacatacata	3704404	43	0.1	0.998	NA	NA
BC3741		methylated-DNA--protein-cysteine S-methyltransferase	tatgaatacatacata	3704404	43	0.5	0.311	NA	NA
BC3742		DNA-3-methyladenine glycosidase	tatgaatacatacata	3704404	43	-1.1	0.029	0.4	0.278
BC3746		hydrolase. alpha/beta fold family	tatgcaattttgcatc	3710301	146	0.3	0.140	-1.4	0.172

gene	name	Function	PlcR box	Location[a]	Dist[b]	r0[c]	p0[c]	r2[c]	p2[c]
BC3747		sensory box/GGDEF family protein	tatgcaattttgcatc	3710301	237	-0.1	0.998	NA	NA
BC3761	*plcA*	1-phosphatidylinositol phosphodiesterase	tatgaaatattgcata	3725577	87	-2.2	0.000	-1.1	0.001
BC3762	*Sfp*	serine protease. subtilase family	tatgcaatatttcata	3727637	144	-3.5	0.004	-1.5	0.003
BC3763	*cwh*	unannotated cell-wall hydrolase	tatgcaatatttcata	3727637	151	NA	NA	NA	NA
BC4072	*sigF*	RNA polymerase sigma-F factor	catgaataattgcatt	4043452	291	NA	NA	-0.3	0.368
BC4142		DNA-damage-inducible protein P. putative	tatgaatttcttcata	4109378	522	0.4	0.184	-1.0	0.193
BC4346		conserved hypothetical protein	tatgcaattttgcata	4286809	504	-0.2	0.156	0.1	0.719
BC4496		glutamate racemase	tatgaaaattttcatt	4442741	82	-0.1	0.248	0.5	0.104
BC4508		rrf2 family protein	tatgcaaatgttcata	4456098	265	0.0	0.458	0.5	0.165
BC4509		Sodium export permease protein	tatgcaaatgttcata	4456098	265	-1.4	0.008	-0.2	0.336
BC4510		ABC transporter. ATP-binding protein	tatgcaaatgttcata	4456098	265	-1.8	0.000	-1.5	0.000
BC4511		acid phosphatase	tatgcaaatgttcata	4456098	62	-3.5	0.000	-1.4	0.023
BC4794		Spore germination protein PF	tatgcacaattgcata	4723091	468	NA	NA	NA	NA
BC4795		conserved domain protein	tatgcacaattgcata	4723091	198	-0.8	0.002	-0.1	0.966
BC4956		Ribosomal-protein-alanine acetyltransferase	tatgcagaattacata	4870034	74	NA	NA	NA	NA
BC4958		NAD(P)H dehydrogenase. quinone family	tatgcagaattacata	4870034	534	0.0	0.999	0.2	0.157
BC4979		conserved hypothetical protein	gatgaattaattcatc	4893912	634	-0.1	0.353	0.7	0.030
BC4980		nifU domain protein	gatgaattaattcatc	4893912	634	0.0	0.330	0.8	0.136
BC4981		aminotransferase. class V	gatgaattaattcatc	4893912	634	0.0	0.947	0.7	0.145
BC4982		conserved hypothetical protein	gatgaattaattcatc	4893912	634	-0.1	0.096	1.0	0.061
BC4983		ABC transporter. ATP-binding protein	gatgaattaattcatc	4893912	634	0.0	0.663	1.4	0.006
BC4984		ABC transporter. substrate-binding protein. putative	gatgaattaattcatc	4893912	634	0.5	0.313	-0.4	0.282
BC4985		ABC transporter. substrate-binding protein. putative	gatgaattaattcatc	4893912	634	0.3	0.385	-0.6	0.112
BC4986		ABC transporter. permease protein	gatgaattaattcatc	4893912	634	1.2	0.039	-0.6	0.172
BC4999		CAAX amino terminal protease family	tatgcaattttacata	4902529	89	-1.3	0.001	0.2	0.750
BC5093		Xanthine permease	tatgtaggatttcata	4998240	367	-0.2	0.720	0.0	0.989
BC5101	*clO*	thiol-activated cytolysin, *clO*	tatgcaatattacata	5004551	91	-5.7	0.000	-4.6	0.000
BC5101a	*sppc1*	small peptide PlcR-controlled	tatgcaatattacata	5004551	91	NA	NA	NA	NA
BC5286		Transcriptional regulator with ABC transporter ATP-binding domain and LytTR DNA-binding domain	tatggaagtttacata	5198202	285	NA	NA	NA	NA
BC5287		stage II sporulation protein D	tatggaagtttacata	5198202	285	0.4	0.998	NA	NA
BC5335	*fbaA*	fructose-bisphosphate aldolase. class II	tatgtatatctacata	5243458	77	0.2	0.382	0.4	0.070

gene	name	Function	PlcR box	Location[a]	Dist[b]	r0[c]	p0[c]	r2[c]	p2[c]
BC5349	*papR*	PapR protein	tatgcaattatgcata	5260284	55	1.0	0.000	2.0	0.000
BC5350	*plcR*	Transcriptional activator plcR	tatgaaataatgcata	5261256	81	3.6	0.000	3.8	0.000
BC5351	*nprB*	Bacillolysin	tatgaaataatgcata	5261256	71	-2.8	0.000	-3.9	0.000
BC5359		aminopeptidase. putative	tatgcaaatatgcata	5278003	119	-0.1	0.373	0.1	0.562
BC5445		superoxide dismutase. Mn	gatgaattaatgcatt	5369359	487	0.2	0.788	-0.3	0.414
BC5448		UDP-glucose 4-epimerase	catgaaaaatttcatg	5373858	243	0.0	0.998	0.2	0.001

Table S1: Microarray results for genes with a PlcR box in their promoter region.

Sequences retained as PlcR boxes were 'TATGNANNNNTNCATA' or ATGHAWWWWTWCAT'. PlcR boxes located more than 700bps and less than 35bps from the open reading frame were discarded. a: 'Location' means PlcR box location on the ATCC14579 chromosome. b: 'Dist.' is the distance between the PlcR box and the first ORF of the putative transcript. c: 'r0' and 'r2' are log2 ratio of gene expression between the PlcR mutant and the wild type strain; r0 was measured at the onset of stationary phase while r2 was measured 2 hours after the entry in stationary phase; p0 and p2 are fdr-corrected p-values, respectively for r0 and r2. Microarray results were missing for Bc0361a, BC2463a, BC3185a, BC3763 and BC5101a because these genes were not included in the microarray chips as they are not annotated in the ATCC14579 sequenced genome. NA means results not available.
