# Supplementary material for: The PlcR Virulence Regulon of Bacillus cereus
Source: PLoS One. 2008 Jul 30;3(7):e2793. doi: 10.1371/journal.pone.0002793 (PMC2464732; doi:10.1371/journal.pone.0002793)
Supplement: Table S2 — Proteins identified in the culture supernatant of the delta-PlcR ATCC14579 strain harvested at t2 (0.19 MB PDF) [file pone.0002793.s002.pdf]

Protein	Function	gene	Mw[a]	pI[a]	Wtype[b]	Localisation[c]	Seq cov[d]	N-terminal sequence	SP[e]
Glycolysis									
G6pA	Glucose 6 phosphate isomerase	BC4898	48	5.0		cytoplasm	14%		No
FbaA	Fructose 6 phosphate aldolase	BC5335	28	5.2		cytoplasm	ND	XLVXMKXML	No
Eno	Enolase	BC5135	44-45	4.5-4.9 & 5.8	■	cytoplasm	31% - 10%		No
Tricarboxylic acid cycle									
CitB	Aconitate hydratase	BC3616	106	4.9	■	cytoplasm	28%		No
FumA	Fumarate hydratase	BC0466	58	5.3		cytoplasm	10%		No
CitC	Isocitrate deshydrogenase	BC4593	39-40	4.9 & 5.2		cytoplasm	10%		No
MdH	Malate deshydrogenase	BC4592	32	5.1		cytoplasm	8%		1-30
PdhA	Pyruvate deshydrogenase alpha subunit	BC3973	36	5.6		membrane	21% - 4%		No
PdhB	Pyruvate deshydrogenase beta subunit	BC3972	33	4.5	■	membrane	20% - 4%		No
Metabolism of other carbohydrates									
Tkt	Transketolase	BC3682	76	4.9		cytoplasm	7% - 3%		No
TreA	Trehalose 6 phosphate hydrolase	BC0632	73	5.0		cytoplasm	17%		No
YvyH	N-acetyl glucosamine epimerase	BC5201	35	5.7		cytoplasm	13% - 4%		No
Amino acid metabolism									
GlyA	Serine hydroxymethyl transferase	BC5316	41	6.0		cytoplasm	9%		No
LeuDH	Leucine deshydrogenase	BC4162	40	5.4		cytoplasm	18% - 3%		No
AlaDH	Alanine deshydrogenase	BC0592	38	5.5 & 5.7		cytoplasm	24%		No
DapD	Tetrahydrodipicolinate succinylase	BC3981	23	5.5		cytoplasm	5%		No
RocA	Pyrroline carboxylate deshydrogenase	BC0344	48-52	5.3-5.7		cytoplasm	20% - 4%		No
Nucleic acids metabolism									
Ndk	Nucleoside diphosphate kinase	BC1515	17	5.5	■	cytoplasm	7%		No
SmbA	Uridylate kinase	BC3823	23	5.7		cytoplasm	6%		No
Pdp	Pyrimidine nucleoside phosphorylase	BC4085	47	4.6		cytoplasm	11%		No
HprT	Hypoxanthine guanilate phosphorybosil transferase	BC0071	20	5.1		cytoplasm	3%		No
GTPase	GTPase	BC2005	18	5.0		membrane	ND	TEEQIIEAV	No
ATP_NADk	ATP-NAD kinase	BC4642	25	6.2		cytoplasm	10%		No
PnP-I	Purine-nucleotide phosphorylase I	BC4086	24	5.1		cytoplasm	9% - 2%		No
Proteins and RNA synthesis									
Ef-G	Elongation factor G	BC0128	86-88	4.9-5.0		cytoplasm	5%	DAGKTTATERIL	No
EF-Ts	Elongation factor T	BC3824	31-32	5.2-5.4		cytoplasm	15% - 5%		1-56
GreA	Transcription elongation factor	BC4374	18	4.7		cytoplasm	8% - 6%		No
Protein folding and general stress proteins									
Tig	Trigger factor (prolyl isomerase)	BC4480	52	4.3		cytoplasm	21% - 3%	KXXKLXXXV	No
GroEL	60 kDa chaperone	BC0295	61	4.8	■	cytoplasm	1%	KXIKFSXXA	No
TerD	Tellurium resistance protein terD	BC0443	21	4.4-4.6 & 5.3		cytoplasm	13%		No
GroES	10kDa chaperone	BC0294	15	4.4		cytoplasm	2%		No

CspD	Cold shock protein D	BC4859	15	4.5 & 4.7	■	cytoplasm	ND	MQXGKVKXFN	No
Detoxification									
SodM	Superoxide dismutase manganese-dependent	BC5445	21	5.3	■	cytoplasm	5%		No
Tpx	Thiol peroxidase	BC4639	18	4.8		cytoplasm	10%		No
AhpF	Alkyl hydroperoxide reductase subunit F	BC0376	52	4.8		membrane	20%		No
AhpC	Alkyl hydroperoxide reductase (C22)	BC0377	20	4.7		cytoplasm	12% - 3%		No
Ribosomal proteins									
RpsF	30S ribosomal protein S6	BC5476	16	5.6		cytoplasm	19% - 6%		No
Mobility and chemotaxis									
FlgA	Flagellin A	BC1657	22-25	4.6-5.2	■	Flagellar	21% - 6%		No
FlgB	Flagellin B	BC1658	23-25	4.5-5.0	■	Flagellar	15% - 4%		No
FlgC	Flagellin C	BC1659	22-24	4.5-5.0	■	Flagellar	21% - 6%		No
FliD	Flagellar hook associated protein 2	BC1638	43	6.5		Flagellar	12% - 6%		No
Membrane proteins (transport, binding and bioenergetics)									
DppE	Dipeptide binding protein	BC1179	57	6.4	■	membrane	29% - 7%		1-26
OppA	Oligopeptide-binding protein OppA	BC1185	55	6.1	■	membrane	16%		1-31
PtgA	PTS system, glucose-specific IIA component	BC5320	18	5.0 & 5.5		membrane	7%		No
Cell wall proteins and endolysins									
LytE	N-acetylmuramoyl-L-alanine amidase	BC5196	48	5.9		membrane	10% - 5%		1-61
CwlA	N-acetylmuramoyl-L-alanine amidase	BC0888	20	6.2		membrane	13% - 2%		1-31
PlyBa	N-acetylmuramoyl-L-alanine amidase	BCp0021	20	6.5		cytoplasm	8%		No
MurA	N-acetyl glucosamine carboxyvinyl transferase	BC5288	40	5.9		membrane	16%		No
MdoB	Phosphoglycerol transferase	BC5232	40	5.2	■	membrane	19% - 13%		No
CwBP	Cell wall-binding protein	BC0679	51	5.9		extracellular	9% - 8%		1-26
CwlB	Cell wall endopeptidase, family M23/M37	BC3698	24	7.0	■	cytoplasm	14%		No
PbpA	Penicillin binding protein	BC4270	73	5.6		membrane	20%		1-35
Unknown proteins									
Unknown	Phage protein	BC1894	34-35	5.5-5.7 & 6.6		membrane	7%		1-39
Unknown	Unknown (homologue pXO2 hypothetical protein)	BC3699	28	7.0		cytoplasm	14%		No
Unknown	Unknown (hypothetical cytosolic protein)	BCp0018	28	5.4	■	cytoplasm	7%		No
Unknown	Unknown	BC1862	27	4.4		cytoplasm	10%		No
Unknown	Unknown	BC2186	19	4.8		cytoplasm	10%		1-19
Unknown	Phage protein	BC1863	16	4.4		cytoplasm	4%		No
Unknown	Unknown (hypothetical cytosolic protein)	BCp0009	22	6.3		cytoplasm			No

Table S2: Proteins identified in the culture supernatant of the delta-PlcR ATCC14579 strain harvested at t2.

Proteins were identified by peptide mass fingerprint or by N-terminal sequencing on a Coomassie Blue-stained two-dimensional electrophoresis gel. (a): Mw and Ip are the molecular weight and the isoelectric point of the protein as determined on the gel. (b): the presence of the protein in the wild-type strain culture supernatant harvested in the

same conditions is indicated by a dark-filled square ‘■’. (c): subcellular localisation as predicted by PSORT. (d): sequence coverage of the protein in %. (e): presence of a signal peptide
