# Supplementary material for: The PlcR Virulence Regulon of Bacillus cereus
Source: PLoS One. 2008 Jul 30;3(7):e2793. doi: 10.1371/journal.pone.0002793 (PMC2464732; doi:10.1371/journal.pone.0002793)
Supplement: Table S3 — Primers used for the directed mutagenesis of the PlcR box (0.04 MB PDF) [file pone.0002793.s003.pdf]

Primer name	5' - 3' sequence [a]	Restriction site	Primer characteristics
OVG	CGTAATCTTACGTCAGTAACTTCCACAGTA		Complementary to *lacZ* 5' end
UP	CGCCAGGGTTTTCCCAGTCACGAC		Universal primer
Bc-plc	TGCTCTAGAGCTCCATGGTCCATTTG	*Xba*I	Complementary to *plcA* 5' end
pRX0	CCCAAGCTTCTATGCAATATTTCATATTG	*Hin*dIII	PlcR box upstream from *plcA*
pRX1	CCCAAGCTTC**A**ATGCAATATTTCATATTG	*Hin*dIII	PlcR box with mutation T_{1A}
pRX2	CCCAAGCTTC**C**ATGCAATATTTCATATTG	*Hin*dIII	PlcR box with mutation T_{1C}
pRX3	CCCAAGCTTC**G**ATGCAATATTTCATATTG	*Hin*dIII	PlcR box with mutation T_{1G}
pRX4	CCCAAGCTTCT**G**TGCAATATTTCATATTG	*Hin*dIII	PlcR box with mutation A_{2G}
pRX5	CCCAAGCTTCTA**C**GCAATATTTCATATTG	*Hin*dIII	PlcR box with mutation T_{3C}
pRX6	CCCAAGCTTCTAT**A**CAATATTTCATATTG	*Hin*dIII	PlcR box with mutation G_{4A}
pRX7	CCCAAGCTTCTATG**G**AATATTTCATATTG	*Hin*dIII	PlcR box with mutation C_{5G}
pRX8	CCCAAGCTTCTATGC**G**ATATTTCATATTG	*Hin*dIII	PlcR box with mutation A_{6G}
pRX9	CCCAAGCTTCTATGC**C**ATATTTCATATTG	*Hin*dIII	PlcR box with mutation A_{6C}
pRX10	CCCAAGCTTCTATGC**T**ATATTTCATATTG	*Hin*dIII	PlcR box with mutation A_{6T}
pRX11	CCCAAGCTTCTATGCA**G**TATTTCATATTG	*Hin*dIII	PlcR box with mutation A_{7G}
pRX12	CCCAAGCTTCTATGCA**C**TATTTCATATTG	*Hin*dIII	PlcR box with mutation A_{7C}
pRX13	CCCAAGCTTCTATGCA**T**TATTTCATATTG	*Hin*dIII	PlcR box with mutation A_{7T}
pRX14	CCCAAGCTTCTATGCAA**G**ATTTCATATTG	*Hin*dIII	PlcR box with mutation T_{8G}
pRX15	CCCAAGCTTCTATGCAA**C**ATTTCATATTG	*Hin*dIII	PlcR box with mutation T_{8C}
pRX16	CCCAAGCTTCTATGCAATATTTCAT**C**TTG	*Hin*dIII	PlcR box with mutation A_{16C}
pRX17	CCCAAGCTTCTATGCAATATTTCAT**G**TTG	*Hin*dIII	PlcR box with mutation A_{16G}
pRX18	CCCAAGCTTCTATGCAATATTTCAT**T**TTG	*Hin*dIII	PlcR box with mutation A_{16T}

Table S3: Primers used for the directed mutagenesis of the PlcR box

[a] The restriction sites are underlined, the wildtype PlcR box is underlined twice, and the modified nucleotides are in bold.
