# Supplementary material for: The PlcR Virulence Regulon of Bacillus cereus
Source: PLoS One. 2008 Jul 30;3(7):e2793. doi: 10.1371/journal.pone.0002793 (PMC2464732; doi:10.1371/journal.pone.0002793)
Supplement: Table S4 — Primers for transcriptional fusions (0.05 MB PDF) [file pone.0002793.s004.pdf]

Gene	Forward primer	Reverse primer
Bc0069[a]	AACTGCAGCTGGCTAGAGCGTACGG	GCTCTAGAGCTAAAAATGCTAGCGGC
Bc0361a[a]	AAACTGCAGCACGCTATTCGCTTTAC	TGCTCTAGATCAGCGTTAAGACTAG
Bc0442[a]	AAACTGCAGGAGTTGTATGATAAAGGAGATCGG	GGTCTAGACAATTGGATCCCAGCCTAAG
Bc0556[a]	AACTGCAGCAGCCAAAATAATCATTGTAGTC	GCTCTAGATAAAGCCATTGTACTAATGCTAAGC
Bc0576[a]	AAACTGCAGGCGCCAAATAAATTAGGACG	GCTCTAGAATGTCTGCAAAGTACGTCGC
Bc0670[a]	AAACTGCAGTGGGATTGGACTAGTGTTTGG	GCTCTAGACTTCAGCAGACCAGCGC
Bc0991[a]	AAACTGCAGGTTTCAACGGACCAATCAAATGTC	GGTCTAGACCTTGTTTATTCGGTACTAACACTCGA
Bc1082[a]	AAACTGCAGCTATGGATAGAATTTTTTCATAATCAAA	GGTCTAGAGCATCAGTCTCTTTTACTAACCGT
Bc1111[a]	AAACTGCAGTATCTGTTACAACTTGTGACGTCG	GGTCTAGACCTATCGTTCGCAGAAATGG
Bc1641[a]	AAACTGCAGCGTTTACATAAAGAACTCCGC	GCTCTAGAGCAAGTGCATTTCATTCG
Bc1713[a]	AAACTGCAGCTTGTACAAATGTATGC	TGCTCTAGATGACGTGGAATGGAT
Bc2410[a]	AAACTGCAGACTTGCTCATATAAATCACCC	GCTCTAGATAAGAAACAAACGCTCAGC
Bc2466[a]	AACTGCAGCAAGCTAAGTTAGCATGTTTAATG	GCTCTAGATCCATATTTGGCGAACAAAG
Bc2707[c]	CCCAAGCTTCGCGCTTATAATAAGGAGGAC	CGGGATCCATCAGTTTCTGTCGCCTTCTC
Bc3384[a]	AAACTGCAGGAGACTTGGCTGAGCAATCTAAAG	GGTCTAGAGTTCGTTCATCAGCATGTAAGTGT
Bc3385[a]	AAACTGCAGTTATACATCAATGATGGGTTCCCC	GGTCTAGACCCCAAAACCCTATTAGGGATAAT
Bc3520[c]	CCCAAGCTTTGGGCAGAACATTAAAGC	CGGGATCCGCTTTATTTGCCCCAATCTTTC
Bc3521[c]	CCCAAGCTTTGTATAGCTACATGAGGATTTTGAC	CGGGATCCCCAAACCCTGTCCGAAAG
Bc3528[b]	CCCAAGCTTTACGAAAGAAGTTACAGCCTCACC	GGTCTAGAGCCAGAATACGTGATAGCTAAGG
Bc3740[a]	AAACTGCAGGCTCTTCAATGCCTAAACCATATG	GGTCTAGACGGTATTCTTGATTTGCATGATGG
Bc3747[a]	AAACTGCAGCGCAGCCATGTATGAT	TGCTCTAGACGAAGCGTACATCTGAAT
Bc3763[a]	AAACTGCAGCACTATTGATAGTGCCTCCTGTTC	GGTCTAGAGTGACATTGTAACGCTTTGCTATC
Bc4142[a]	AAACTGCAGCGCATTGACTCTGAAACGAC	GGTCTAGAACTCACTCCCTAATCAGAACGTT
Bc4510[a]	AAACTGCAGCTTCAGCCTCAGCTTTGTTAATCC	GGTCTAGACCTAGTACTTCACCTTTTGGTAACGAA
Bc4511[a]	AAACTGCAGCCCATGTAATCGAACCTTCATTAG	GGTCTAGAGCTGTTTGATACCATAAATCAGCC
Bc4794[a]	AACTGCAGCGTAGGACATCACTATCTGAGTTC	GCTCTAGATCCATTATAAGCCTTCGTCTTTTC
Bc4795[a]	AAACTGCAGCAATAATAACACCTTCCACAACTGCC	GGTCTAGAGTCGTCCTTCTCGCTTCATTAAAAT
Bc4958[a]	AAACTGCAGAACTATAAGGTTCCTCGTACGTCC	GGTCTAGACGTGACCTTTTCCTTCTAATTCAC
Bc4986[a]	AACTGCAGATTGTTTAATCAGTTAGAGAAACC	GCTCTAGATTGCCGTCATATATAACGTTTC
Bc5335[a]	AAACTGCAGGTCAAGCTGTGAGAAATGAGC	GCTCTAGAATTTTTCTTCTTCCGCAGC
Bc5359[a]	AAACTGCAGAGATGGAAAAATCATCCGAGAAGG	GGTCTAGACCCATTGATTTAAGCTGCATACCA

Table S4: Primers for transcriptional fusions.

Primers used to amplify promoter regions of genes selected for lacZ fusions are given in the table. Restriction sites are underlined and were PstI and XbaI (a), HindIII and XbaI (b), or HindIII and BamHI (c).
