# Supplementary material for: The PlcR Virulence Regulon of Bacillus cereus
Source: PLoS One. 2008 Jul 30;3(7):e2793. doi: 10.1371/journal.pone.0002793 (PMC2464732; doi:10.1371/journal.pone.0002793)
Supplement: Figure S1 — Results from lacZ fusions (0.13 MB PDF) [file pone.0002793.s005.pdf]

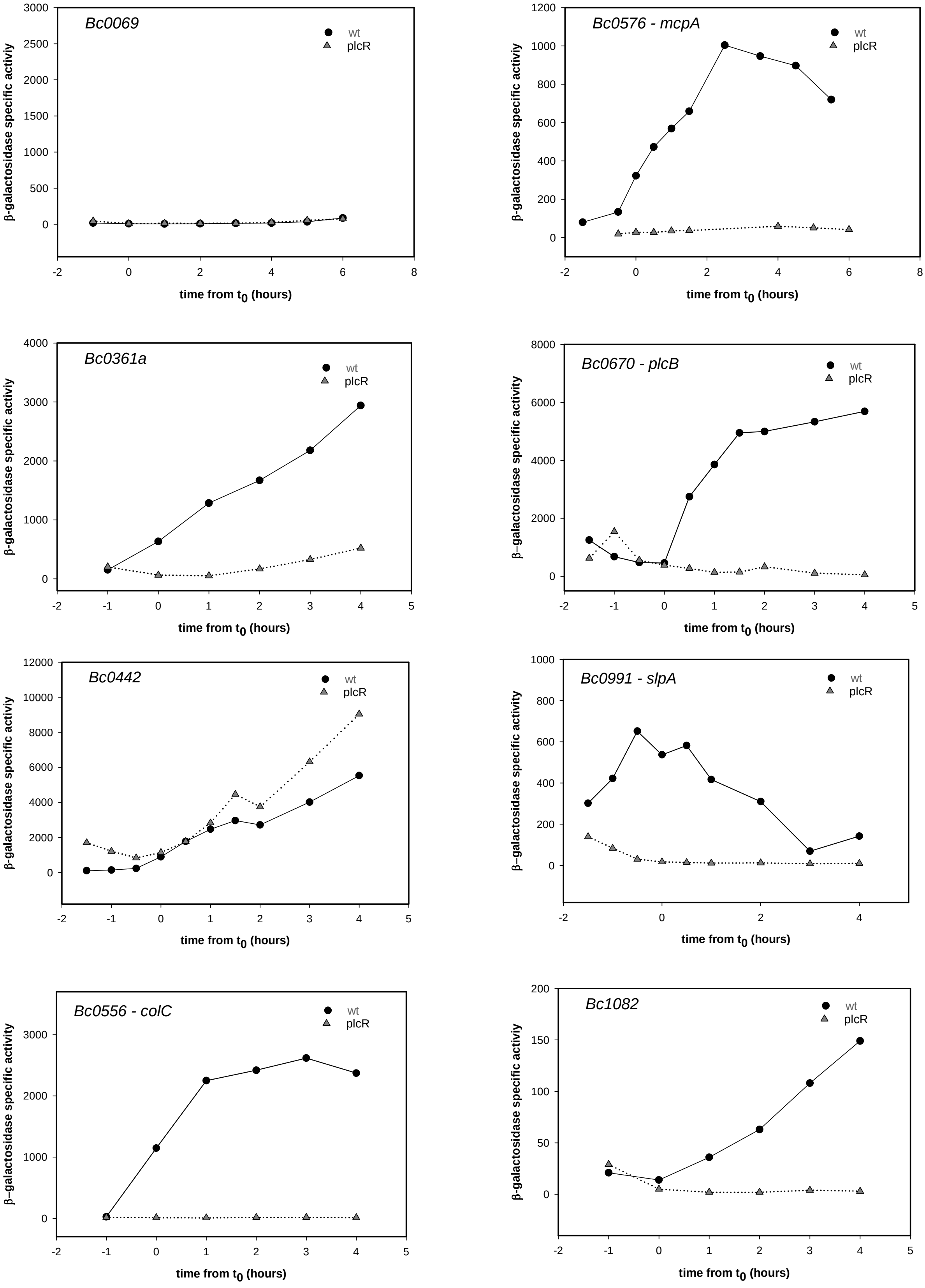
Bc0069
Bc0576 - mcpA
Bc0361a
Bc0670 - plcB
Bc0442
Bc0991 - slpA
Bc0556 - colC
Bc1082
wt
plcR
β-galactosidase specific activiy
β-galactosidase specific activity
time from t_0 (hours)

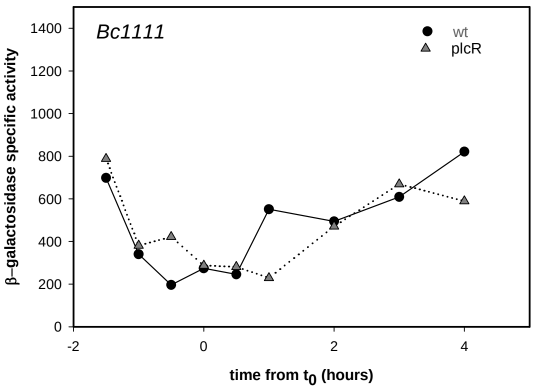
Bc1111
wt
plcR
β-galactosidase specific activity
time from t0 (hours)

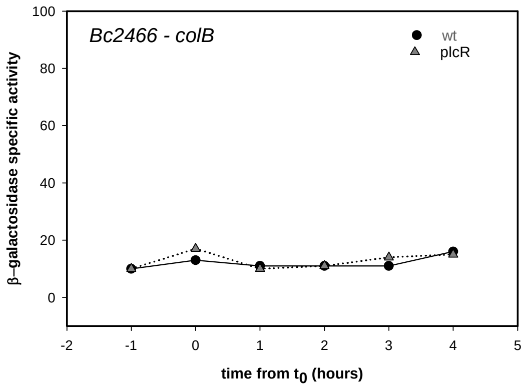
Bc2466 - colB
wt
plcR
β-galactosidase specific activity
time from t0 (hours)

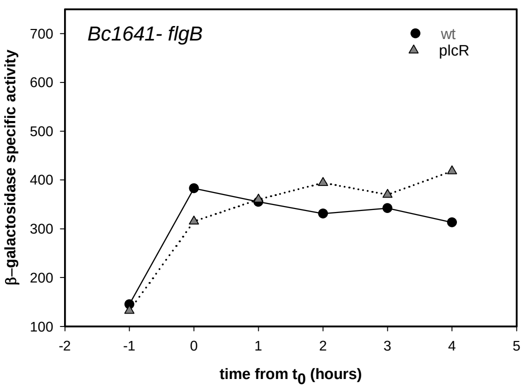
Bc1641- flgB
wt
plcR
β-galactosidase specific activity
time from t0 (hours)

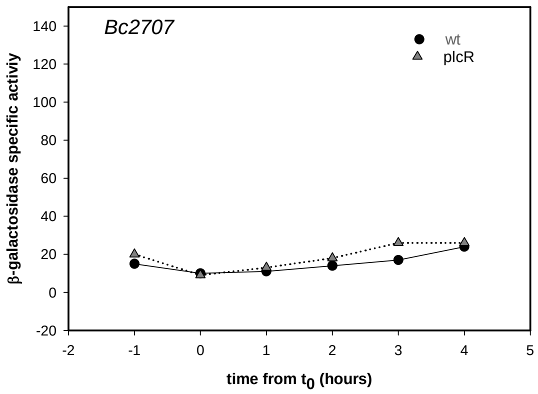
Bc2707
wt
plcR
β-galactosidase specific activiy
time from t0 (hours)

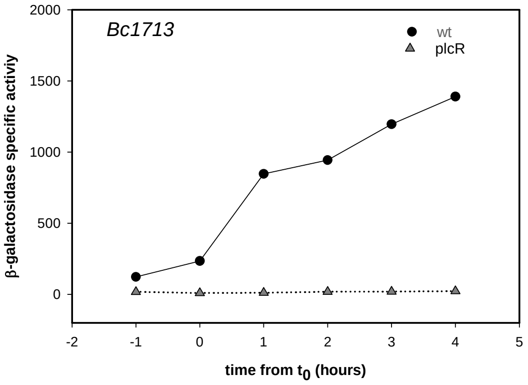
Bc1713
wt
plcR
β-galactosidase specific activiy
time from t0 (hours)

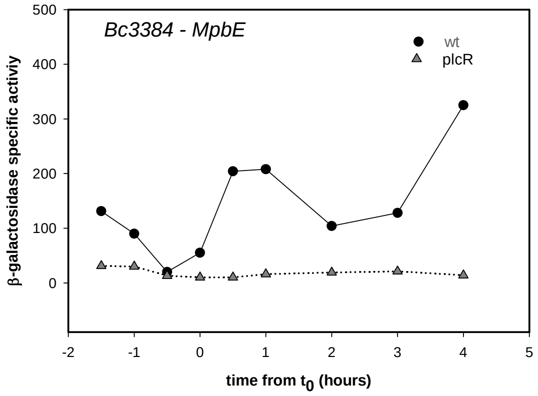
Bc3384 - MpbE
wt
plcR
β-galactosidase specific activiy
time from t0 (hours)

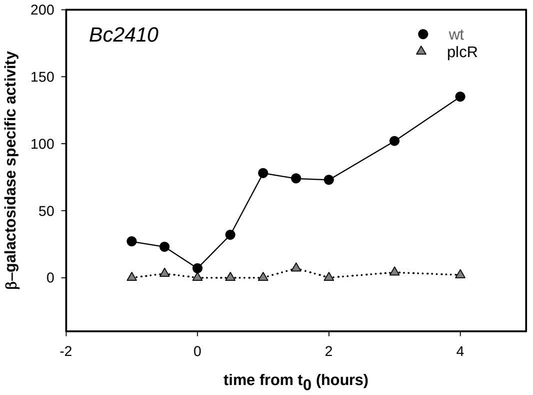
Bc2410
wt
plcR
β-galactosidase specific activity
time from t0 (hours)

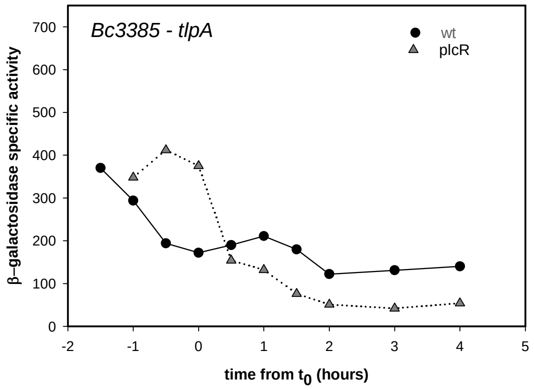
Bc3385 - tlpA
wt
plcR
β-galactosidase specific activity
time from t0 (hours)

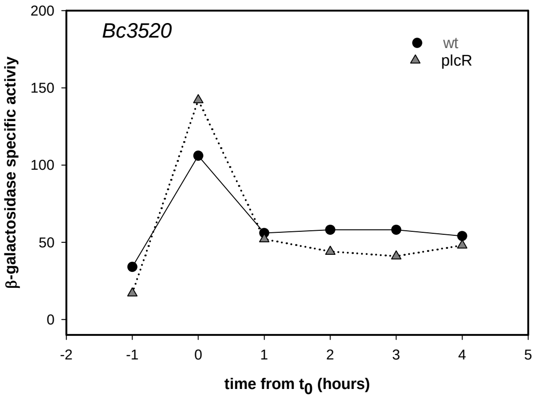
Bc3520
wt
plcR
β-galactosidase specific activiy
time from t0 (hours)
0
50
100
150
200
-2
-1
0
1
2
3
4
5

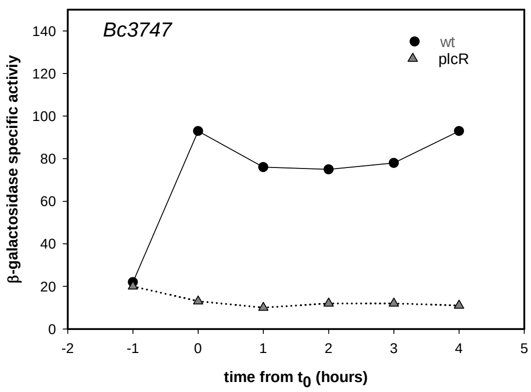
Bc3747
wt
plcR
β-galactosidase specific activiy
time from t0 (hours)
0
20
40
60
80
100
120
140
-2
-1
0
1
2
3
4
5

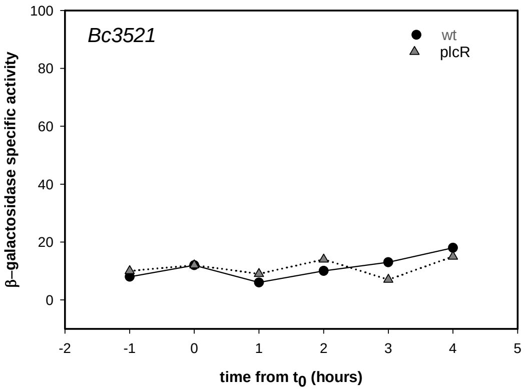
Bc3521
wt
plcR
β-galactosidase specific activity
time from t0 (hours)
0
20
40
60
80
100
-2
-1
0
1
2
3
4
5

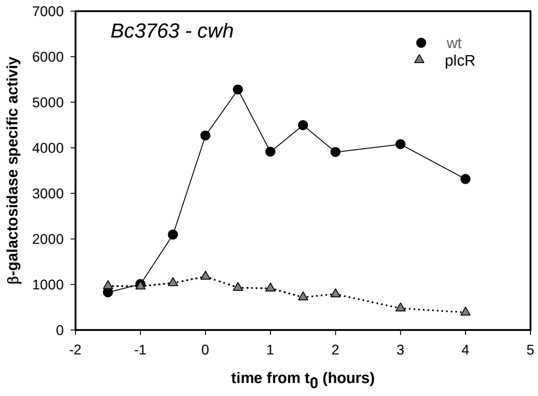
Bc3763 - cwh
wt
plcR
β-galactosidase specific activiy
time from t0 (hours)
0
1000
2000
3000
4000
5000
6000
7000
-2
-1
0
1
2
3
4
5

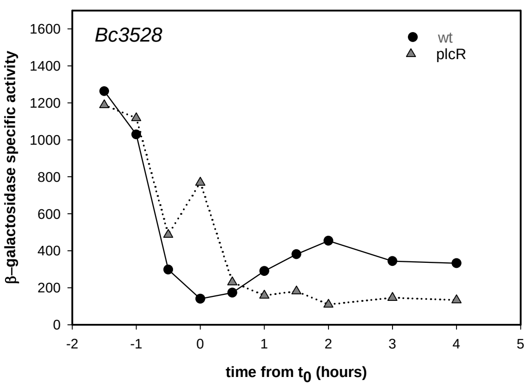
Bc3528
wt
plcR
β-galactosidase specific activity
time from t0 (hours)
0
200
400
600
800
1000
1200
1400
1600
-2
-1
0
1
2
3
4
5

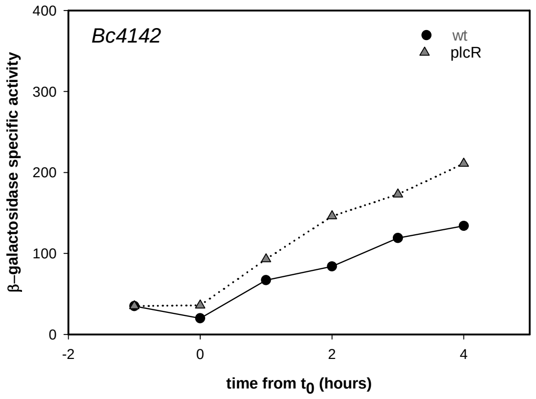
Bc4142
wt
plcR
β-galactosidase specific activity
time from t0 (hours)
0
100
200
300
400
-2
0
2
4

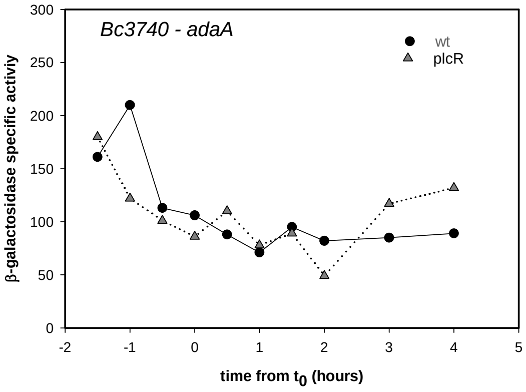
Bc3740 - adaA
wt
plcR
β-galactosidase specific activiy
time from t0 (hours)
0
50
100
150
200
250
300
-2
-1
0
1
2
3
4
5

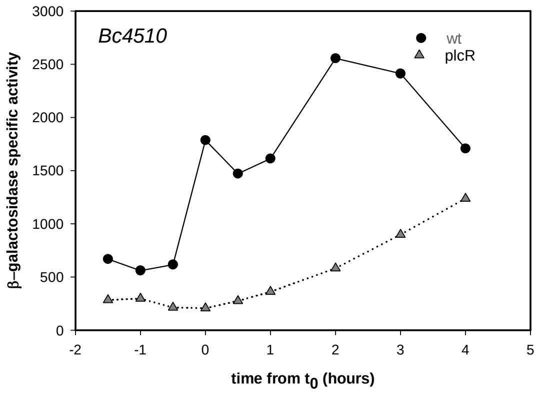
Bc4510
wt
plcR
β-galactosidase specific activity
time from t0 (hours)
0
500
1000
1500
2000
2500
3000
-2
-1
0
1
2
3
4
5

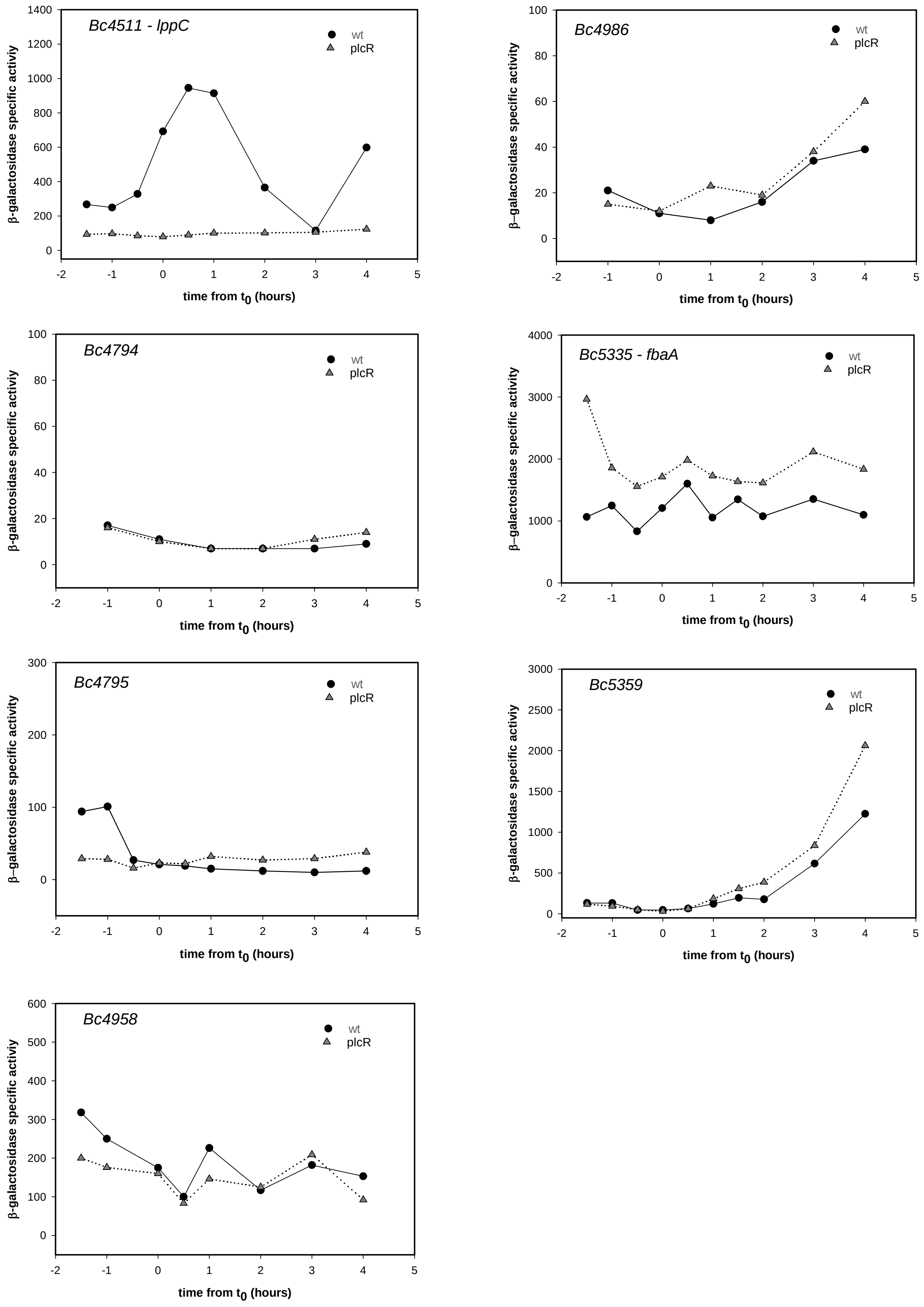

Figure S1: Results from *lacZ* fusions.

Transcriptional fusions were performed between genes promoter regions and *lacZ*. Beta-galactosidase activity is plotted as a function of time of for the ATCC14579 wild type and mutant strains.
