# Supplementary material for: The PlcR Virulence Regulon of Bacillus cereus
Source: PLoS One. 2008 Jul 30;3(7):e2793. doi: 10.1371/journal.pone.0002793 (PMC2464732; doi:10.1371/journal.pone.0002793)
Supplement: Figure S2 — Two-dimensional gel electrophoresis of the Δ-plcR ATCC14579 supernatant (0.97 MB PDF) [file pone.0002793.s006.pdf]

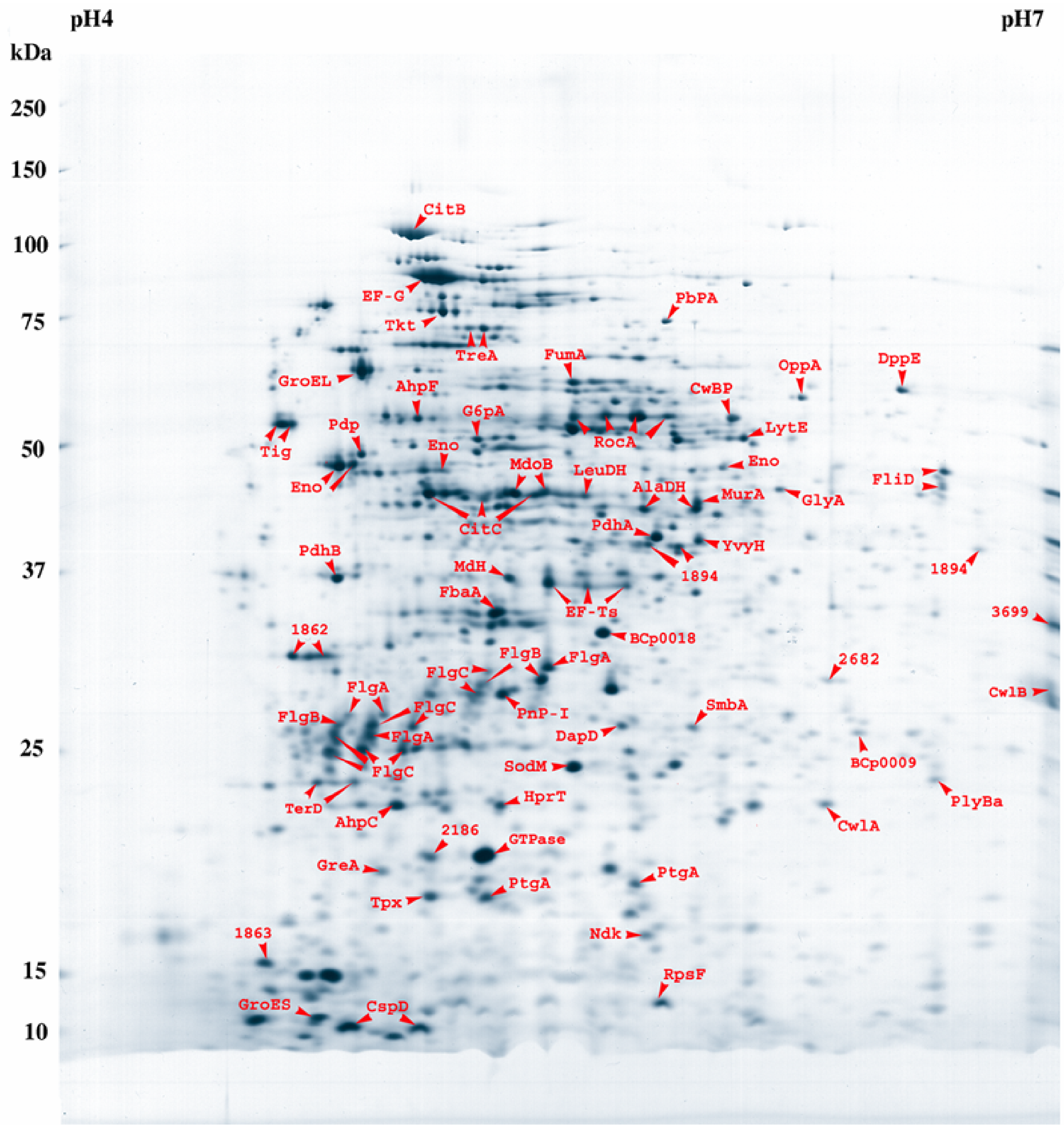

Figure S2: Two-dimensional gel electrophoresis of the Δ-plcR ATCC14579 supernatant.
The supernatant was harvested at t_2. The gel was silver-stained. In red, abbreviated name of the proteins present in the spots, and identified by mass spectrometry or by N-terminal sequencing (see table s3). Top scale: pH range; left scale: MW range
