# Supplementary material for: The PlcR Virulence Regulon of Bacillus cereus
Source: PLoS One. 2008 Jul 30;3(7):e2793. doi: 10.1371/journal.pone.0002793 (PMC2464732; doi:10.1371/journal.pone.0002793)
Supplement: Figure S3 — Location of PlcR boxes on the ATCC14579 chromosome (0.02 MB PDF) [file pone.0002793.s007.pdf]

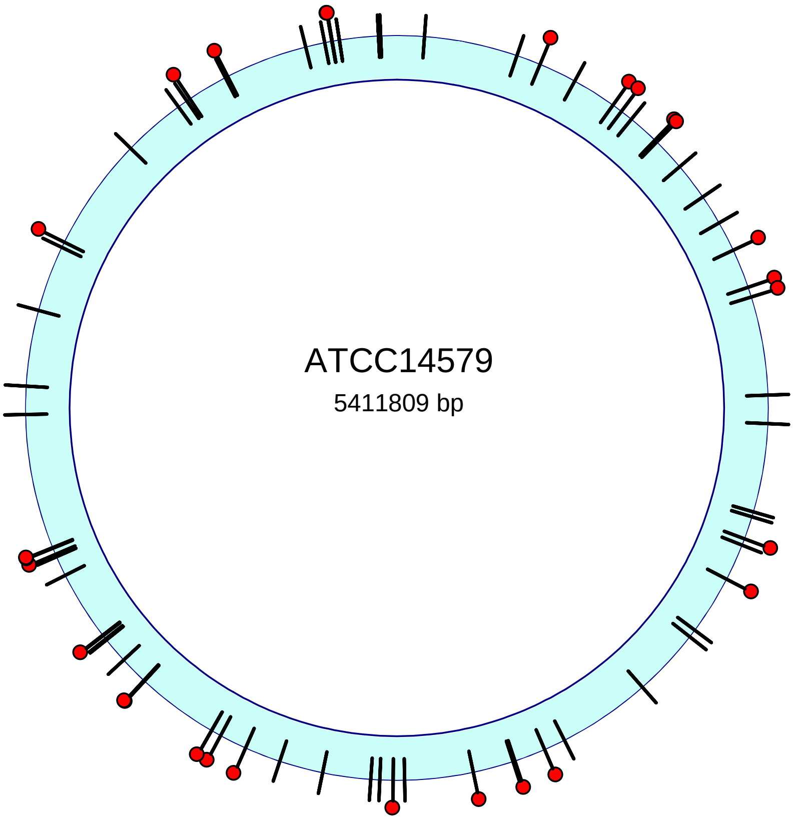

Figure S3: Location of PlcR boxes on the ATCC14579 chromosome.
Each dark line represents a PlcR box (active or inactive) and red circles show active PlcR boxes. The chromosome origin is placed on top of the blue circle.
