# Supplementary material for: The PlcR Virulence Regulon of Bacillus cereus
Source: PLoS One. 2008 Jul 30;3(7):e2793. doi: 10.1371/journal.pone.0002793 (PMC2464732; doi:10.1371/journal.pone.0002793)
Supplement: Figure S4 — Genetic environment of the 45 PlcR-regulated genes (0.26 MB PDF) [file pone.0002793.s008.pdf]

PlcR-controlled genes	Genetic organization
Bc0361a<br>Bc0362	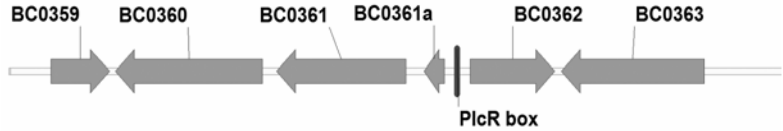
Bc0556	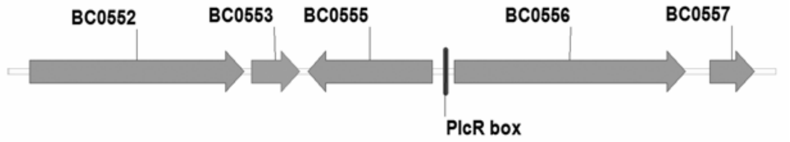
Bc0576<br>Bc0577<br>Bc0578	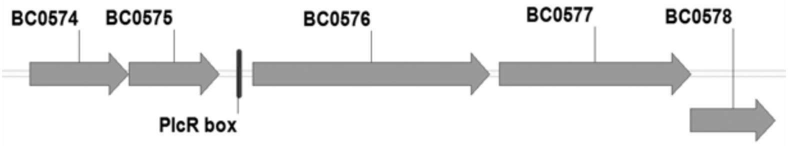
Bc0666	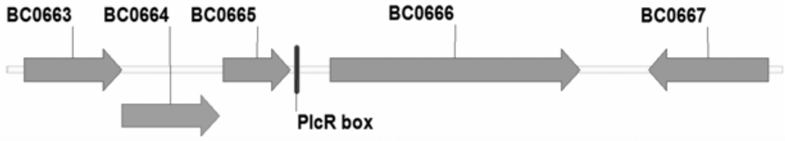
Bc0670<br>Bc0671	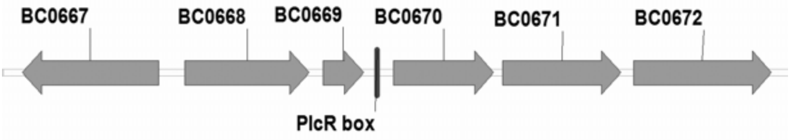
Bc0991	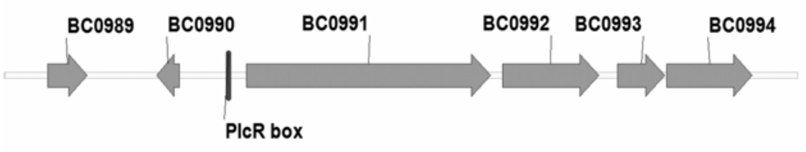
Bc1081<br>Bc1082	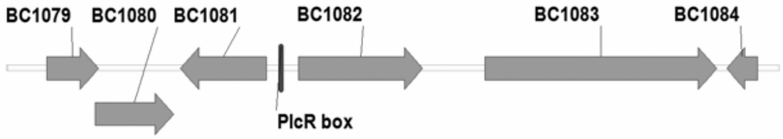
Bc1110	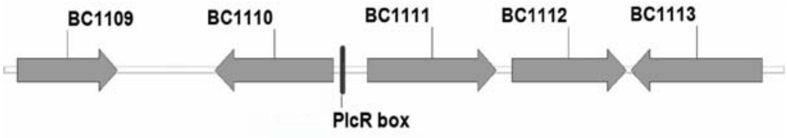
Bc1713	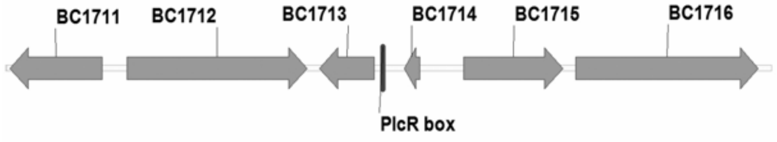
Bc1809<br>Bc1810<br>Bc1811	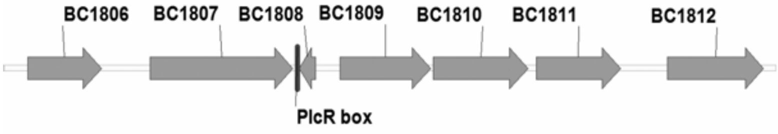
Bc2410<br>Bc2411	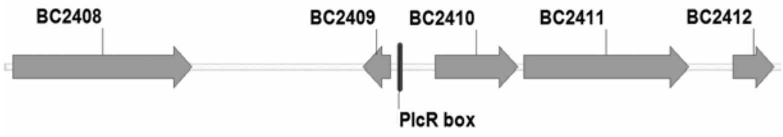
Bc2463a	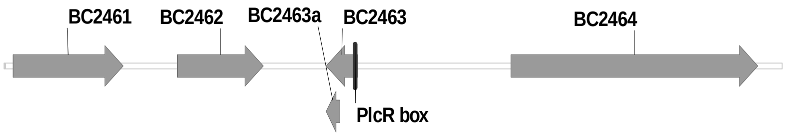

Bc2552

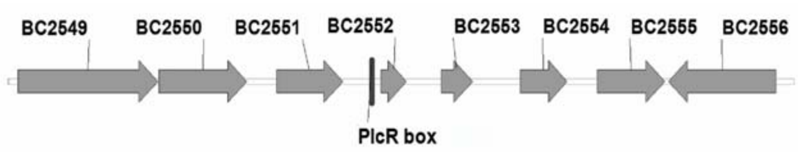

Bc2735

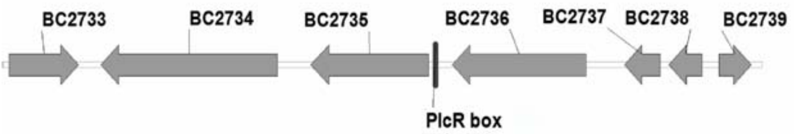

Bc3102
Bc3103
Bc3104

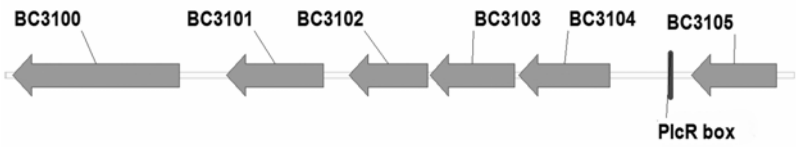

Bc3161

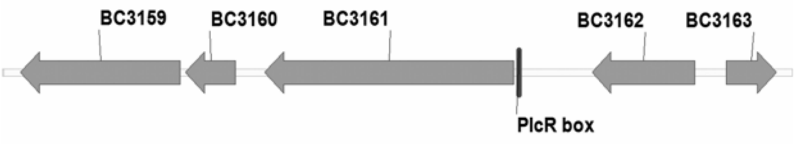

Bc3185a

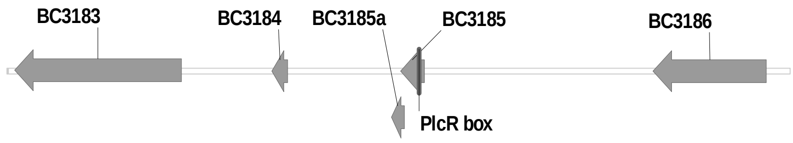

Bc3383
Bc3384
Bc3385

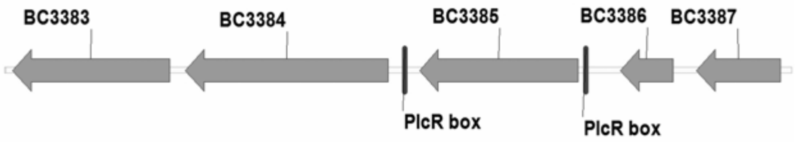

Bc3527

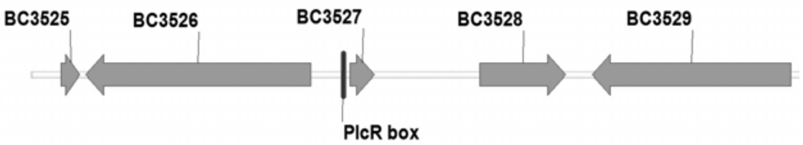

Bc3746
Bc3747

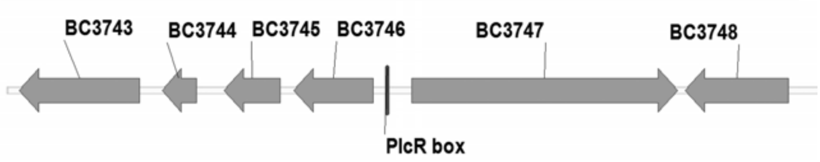

Bc3761
Bc3762
Bc3763

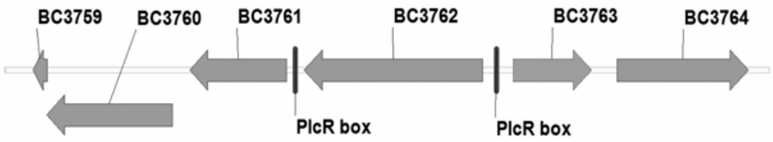

Bc4509
Bc4510
Bc4511

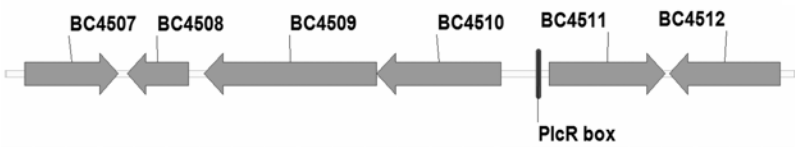

Bc4999

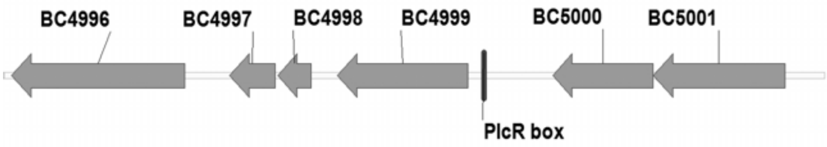

Bc5101
Bc5101a

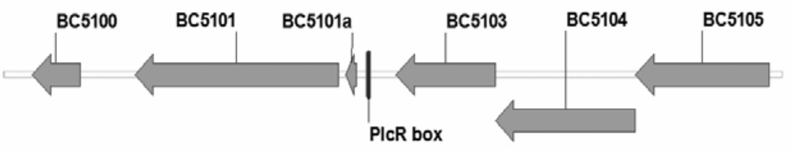

Bc5349
Bc5350
Bc5351

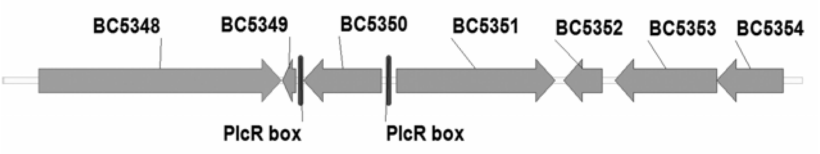

Figure S4: Genetic environment of the 45 PlcR-regulated genes.
