# Supplementary material for: The PlcR Virulence Regulon of Bacillus cereus
Source: PLoS One. 2008 Jul 30;3(7):e2793. doi: 10.1371/journal.pone.0002793 (PMC2464732; doi:10.1371/journal.pone.0002793)
Supplement: Figure S5 — Putative −10 σA boxes located downstream of PlcR boxes for PlcR-controlled genes (0.60 MB PDF) [file pone.0002793.s009.pdf]

AAAAGTACAAAATATGCAATATTACATATTGGAACTCAACAAAATACTCCATTGATAAGTACATAGATCGAACGAAGGCATCGTT	Bc2410
AAAGATTCTCAATATGTAATATTGCATATTGGCCGTTTTATAGTTTTAATATTATTTTTAAAAAGAAAAAGTACGAGGTGATTCA	Bc1082
TTGTGTATATGCTATGCATAATTGCATATGAGTTTAAAAATTATATCGATATAATTTTATCGGTTATTTGTAAGTAGTGTTTTCT	Bc1809
ATAAGGAGTGAATATGCAAAAATGCATAATTGACGAAAAAATTACATGTTATCATTATATATTGTTTTATATATGTTAATTGTAT	Bc0576
TACAAAAGACAATATGCAAATGTTCATATAAAAATATTATATTTTAATATATAATATAAAATGATTTTCTAACATCAAGGAGGAT	Bc4511
TAAAACGGCCAATATGCAATATTACATATTGAGAATCTTTTTACTCTTGATACAATATTCATACATCAAAATAAAAGGAATGTAT	Bc1081
CTAAAAAATGGGTATGCATAATTGCATATAATAGAGATAAATTTTCATGATATATTAAAATAAAAAATGTGGGTGATGGAATATG	Bc5349
TTGACCACAATTTATGTAATTTTGCATAATGTTACATAAATTGTGTATTTTCTCTTTTTTTAGACATTAATAGACAGTATACTTT	Bc2735
TTTTATATATATTATGCATTATTTCATATCAAAAATTGTCGAATTCACATTATTGTAGTGGTATGACAACTCAAAAAATTAGATT	Bc5350
AGCGGAATATATTATGCAATATTGCATTTGATAATCATTTTCACTGTAACTATAATGATTACAGGGTTACTATTAATCCAATATT	Bc3527
AATACATATCCTTATGCAAAATTGCATATTAATATGTATTCTTCTTACTTTAAAATTAGCTTAAATCACTTTCCAAATTATACAA	Bc0361a
AGGAAGAAATAATATGAAATATTGCATTTTATATTGTTACATCCAGATTTTATGTTAAATTCTTGAATGGAATATTAAAATAGGG	Bc0556
TCAAAACTCATCTATGCAATTATGCATAACCATCCATTCCGTTTTATTTTCATGTTACGATATAAATGTAATACGACATATATCG	Bc1110
AAAACGCGCGAATATGCAATTCTGCATATTCACACGTTTTATCACAATGTTATGATTTAATTTAGTTAAAGAACAAGTATACCGA	Bc1713
CTCATCATTTTATATGCAATTTTGCATCTCCACTTACAAATCACTAAAAAAACTTTACAATTCAAAGGATGACTAAATATACAAT	Bc3747
TTGTTAATACGATATGCAAATATGCATATCGTATTTTTTTGAGTTCTTTATGTGTTAAAATTAGATATAAAGATATGAAAATATA	Bc2552
TAAAAGAATTTTCATGCAATTTTGCATATTGTCTTTTAAATTTGTTATATAAGATATTTAATATATATCACATAACTCTTTATTT	Bc0666
GACAATTTTTGATATGAAATAATGCATAATATATATAAAATTCGGCAAATTTTTCATACAATTCATCTCACATACAATAATAGAT	Bc5351
CATGTAATTAAATATGAAATAATACATATTATAATAATTTATTAAGATAAAATGTTATATTTTTGGGTGGAATATATAAATGGAG	Bc3161
ATAATATTTTTATATGAACATTTGCATATTGTCTTTTGTATATATAGTAGTATGTCCTATATTAGAAAGATGAGCAAAATAATAA	Bc4510
AATGTATTTTCATATGCAATATTTCATATTCTGACTCTACACTAAACGAATATGAAACTTATAGTGATTATTTATTTTATATGTG	Bc3763
GTAGAGTCAGAATATGAAATATTGCATATGAAAATACATTGAAGTTCATATTTCTGATTACTATAATCTATTTAGTAGAAAGGGA	Bc3762
AAGTTTCATAATATGTAAAATTGCATATTATTCTTTTTGAAAAAATTGTTATATTATCTATATATTTGTATTAAAGGATTTTTG	Bc4999
CTTGTTTTTTTATATGTAATATTGCATATCAGAGTGTATTGATAAAAGTGTAAGGGGTCACTATAATGAGGGCATAAAGATAAAT	Bc5101a
CTAATGTATAAATATGCAATATTGCATACGGAAATGTATTGTTATTATTGTAAGCGCTTACTATAATAAAGTTATAAAGATTTAT	Bc2463a
AAAACGTATAAATATGCAATATTGCATACAGAAATGTATTGTTACAAATGTAAGCGTTTACTATAATGAAGGTATAAAGTTTAAT	Bc3185a
TTTATATCCATATATGCAAAATTACATATATTTAATGATTGATAATCATGAGCTAATGTAACTATAATAATTACACTAATAAGTA	Bc3385
TTTGTAAGTGGAGATGCAAAATTGCATATAAAATGATGAGTATCTTTTTTTGGTGAGCTTTTTATTTTTAATCTGAAGATTAATA	Bc3746
ATATCTACATTTTATGCAATTATACATAACTAAATAAAGGTAAAAAAGTATAAAAAGACCTATTATATTATTCTATAAGTATTTT	Bc3104
TAACTTTACTTCTATGCAATATTTCATATTGATGAAATGTTATTTATTTTTTCTGAACTAGCTTATATTATATTCAATAAGTTTG	Bc3761
AGTAGTGGATGATATGCAAAATTGCATATTACAAAATAAACATCGTAATTTTTGGATTCTTGGTATAAAATACATTACTTAAACT	Bc3384
AATACATATTAATATGCAATTTTGCATAAGGATATGTATTTTATTAAAATATAAAGATTTTATACTATAATGAAAACCGCTATAA	Bc0362
AAGTTATAATGATATGAACATTTGCATATTTTAATTTATTGATAGAAATTTCATGAAAGGTGGATATTCTAGTCATAGGTTAAC	Bc0670
TCCTTATAACAATATGAATTTTTGCATATTTTTAAATTCAATATATCCCTCAAAAACTTTTAATTGTATAATATATTTTGTCATA	Bc0991

Figure S5: Putative -10 σ^A boxes located downstream of PlcR boxes for PlcR-controlled genes. The presence of possible -35 boxes were considered for the determination of best -10 boxes locations. While there are 28 active PlcR boxes, 34 -10 σ^A boxes are shown because some of the PlcR boxes can work both in the forward and reverse directions.
